# Treatment of oral cancer using magnetized paclitaxel

**DOI:** 10.18632/oncotarget.24570

**Published:** 2018-02-26

**Authors:** Rina Nakakaji, Masanari Umemura, Kenji Mitsudo, Jeong-Hwan Kim, Yujiro Hoshino, Itaru Sato, Takatsugu Masuda, Masahiro Yamamoto, Mitomu Kioi, Toshiyuki Koizumi, Takayuki Fujita, Utako Yokoyama, Masaki Iida, Motohiko Sato, Hiroshi Sato, Shoko Murofushi, Sayaka Shibata, Ichio Aoki, Haruki Eguchi, Iwai Tohnai, Yoshihiro Ishikawa

**Affiliations:** ^1^ Cardiovascular Research Institute, Yokohama City University Graduate School of Medicine, Yokohama, Japan; ^2^ Department of Oral and Maxillofacial Surgery, Yokohama City University Graduate School of Medicine, Yokohama, Japan; ^3^ Department of Environment and Natural Sciences, Yokohama National University Graduate School of Environment and Information Sciences, Yokohama, Japan; ^4^ Tokyo Neutron Science Laboratory, Tokyo University Institute for Solid State Physics, Kashiwa, Japan; ^5^ Department of Chemistry of Functional Molecules, Konan University Faculty of Science and Engineering, Kobe, Japan; ^6^ Department of Physiology, Aichi Medical University, Nagakute, Japan; ^7^ Advanced Applied Science Department, Research Laboratory, IHI Corporation, Yokohama, Japan; ^8^ Molecular Imaging Center, National Institute of Radiological Sciences, Chiba, Japan

**Keywords:** iron-salen, taxol, oral cancer, paclitaxel, magnetism

## Abstract

*N,N’-Bis(salicylidene)ethylenediamine iron* (Fe(Salen)) is an anti-cancer agent with intrinsic magnetic property. Here, we covalently linked Fe(Salen) to paclitaxel (PTX), a widely used anti-cancer drug, to obtain a magnetized paclitaxel conjugate (M-PTX), which exhibited magnetic characteristics for magnet-guided drug delivery and MRI visualization. M-PTX increased apoptosis and G2/M arrest of cultured human oral cancer cell lines in the same manner as PTX. Furthermore, marked contrast intensity was obtained in magnetic resonance imaging (MRI) of M-PTX. In a mouse oral cancer model, a permanent magnet placed on the body surface adjacent to the tumor resulted in distinct accumulation of M-PTX, and the anti-cancer effect was greater than that of M-PTX without the magnet. We believe that this strategy may improve future cancer chemotherapy by providing conventional anti-cancer drugs with novel functionalities such as magnet-guided drug delivery or MRI-based visualization/quantitation of drug distribution.

## INTRODUCTION

Despite advances in multimodality treatment, *e.g.* surgery, chemotherapy, and radiation, the prognosis for patients with oral squamous cell carcinoma (SCC) has remained poor, with the overall 5-year relative survival rate of about 50% [1]. Surgical removal of the cancer is currently the gold standard treatment, but is associated with various complications, such as dysphagia or dysarthria.

To preserve organ and function, platinum-based concurrent chemoradiotherapy or bioradiotherapy using cetuximab represents a definitive treatment modality for locally advanced SCC of the head and neck [2, 3]. However, acute adverse effects, including hematotoxicities, renal failure, mucositis, dysphagia, or nausea/vomiting, often occur in those patients, who received concurrent high dose cisplatin and radiotherapy. Further, osteoradionecrosis of the mandible, as a late adverse event, may also occur in patients with oral SCC. Therefore, we need to improve the efficacy of such treatments and to decrease these adverse effects.

Paclitaxel has been widely used in the chemotherapy as an induction therapy or secondary treatment for recurrent/metastatic SCC of head and neck. The efficacy of the combination therapy, *i.e.*, paclitaxel and other anticancer agent, such as cetuximab, has been reported for the SCC of head and neck [3–5]. However, the toxicity of paclitaxel is occasionally unacceptable. Thus, the development of alternative methods, such a new drug delivery system (DDS), is desired for paclitaxel, as well as other anticancer drugs, to reduce these adverse effects.

In this connection, we have previously reported several pre-clinical studies of *N,N’-bis(salicylidene)ethylenediamine iron* (Fe(Salen)), an anti-cancer agent that also has inherent magnetic character [6–9]. Fe(Salen) offers various advantages as a multimodal anti-cancer agent: it has intrinsic anti-cancer activity; it can be attracted by a magnet for targeted delivery; it can be visualized by magnetic resonance imaging (MRI); and it generates heat when exposed to an alternating magnetic field (AMF), resulting in local cytotoxicity. We have demonstrated the effectiveness of combined hyperthermia-chemotherapy with magnetically guided Fe(Salen) nanoparticles (NPs) to treat tongue tumor in a rabbit model [7]. Intravenous administration of Fe(Salen) NPs *per se* suppressed the tumor growth even before magnetically guided delivery and AMF-induced heating were applied, but addition of these two magneto-responsive modalities dramatically improved the anti-cancer activity, and the tumor mass was greatly reduced. We also evaluated the synergistic anti-cancer and hyperthermia-inducing effects of Fe(Salen) NPs in human glioblastoma (GB: WHO Grade IV) *in vitro* and *in vivo* [8]. The combination of Fe(Salen) local injection and AMF exposure (combined hyperthermia-chemotherapy) showed a greater anti-cancer effect in a mouse back tumor model of GB than did either Fe(Salen) NPs alone or carmustine (BCNU) alone. These findings indicate that combined hyperthermia and chemotherapy with single-drug NPs could be a promising strategy for cancer treatment.

Based on the above findings, we considered that magnetic delivery of other anti-cancer agents would be an effective strategy for enhancing their anti-cancer activity. To achieve this, we hypothesized that covalent linking of Fe(Salen) with another low-molecular-weight anti-cancer drug would endow the combined molecule with intrinsic magnetic character. In the present work, we aimed to test this idea by covalently linking Fe(Salen) to paclitaxel (PTX) [6, 10]. We chose PTX because it is a well-known anti-cancer drug [11] that is effective against various cancers, including head and neck cancers [4]. However, its efficacy and tolerability are restricted by its low solubility and lack of selective tumor uptake, resulting in excessive systemic exposure that leads to side effects such as nausea, vomiting, diarrhea, mucositis, myelosuppression, and cardiotoxicity. Therefore, we considered that if it could be magnetically targeted to the tumor site, this would increase its anti-cancer efficacy while simultaneously reducing systemic side effects.

Here, we report the design and synthesis of a novel class of combined drug, consisting of PTX covalently bound to Fe(Salen) as a molecular component of magnetic nanoparticle, which we designate as magnetized PTX (M-PTX). We emphasize that this M-PTX conjugate system is completely different from a nanocarrier or micellar system in which PTX is encapsulated as a physical mixture with magnetic particles such as iron oxide NPs. We confirmed that M-PTX retains the anti-cancer characteristics of PTX. Further, the anti-cancer effect of magnetically targeted M-PTX is greater than that of either M-PTX or PTX in the absence of the magnet, both *in vitro* and *in vivo*. These results support the validity of our strategy, and suggest that it might be similarly applicable to other conventional anti-cancer agents.

## RESULTS

### Synthesis and characterization of M-PTX

**Figure 1:**
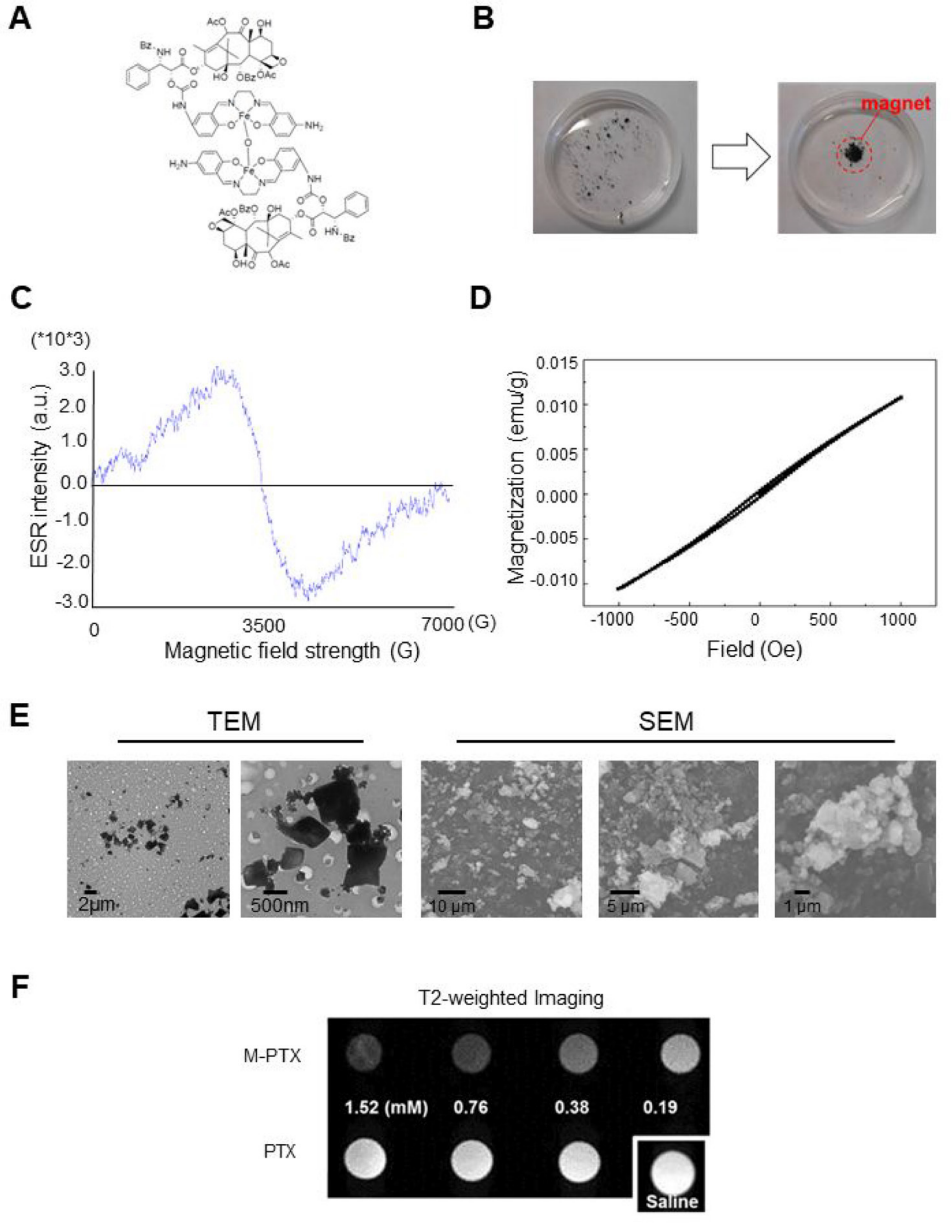
Magnetization of PTX by covalent linking of M-PTX (**A**) Chemical structure of M-PTX prepared by covalent linking of PTX with Fe(Salen). This compound was synthesized and characterized as described in Materials and Methods. Purity was confirmed by means of HPLC and MS (API-ES). (**B**) Magnetic accumulation of M-PTX by a permanent magnet in static water. (**C**) Electron paramagnetic resonance (ESR) spectrum of M-PTX (powder) was measured with an X-band ESR spectrometer (Bruker EMXPLUS 8/2.7S). Microwave power and modulation amplitude were 1 mW and 1 G, respectively. The values of G at the peak convex upward and the convex downward are the same as Fe(Salen) solution (50 mM). M-PTX retained the same magnetic properties of Fe(Salen) solution (50 mM) and core-shell nanoassemblies in terms of the ESR measurement. (**D**) Plots of magnetization versus magnetic field for M-PTX. A plot of magnetization versus magnetic field was generated using a superconducting quantum interference device (SQUID) (Quantum Design MPMS7 system). Measurements were made at 37° C (310 K). Changes in magnetization (M/(emu/g)) with applied magnetic field (H/Oe) between –1,000 and 1,000 Oe are shown. M-PTX particles were stable and retained the same magnetization-versus-magnetic-field curve for at least three years in air. (**E**) Transmitting electron microscope (TEM) and scanning electron microscope (SEM) imaging of M-PTX. A scale bar is shown at the bottom. (**F**) MR imaging of M-PTX. T2-weighted images of PTX and M-PTX (0–1.5 mM) were obtained with a 9.4-Tesla scanner. Note that signal intensity changed in a concentration-dependent manner for M-PTX, but not for PTX alone.

Fe(Salen) was conjugated to PTX as described in Materials and Methods. The M-PTX conjugate spontaneously dimerized via μ*-oxo* bridge formation between the Fe centers (Figure 1A). M-PTX was readily accumulated by a magnet in static and flowing water (Figure 1B and Supplementary Movie 1), as could be easily seen from the brown color derived from the Fe(Salen) residue. It should be noted that PTX(s) and Fe(Salen) covalently linked a number of molecule ratio of 1:1 (M-PTX A), 2:1 (M-PTX B), 3:1 (M-PTX C) and 4:1 (M-PTX D), as shown in Supplementary Figure 1. The products showed only low levels of impurities as determined by mass spectrometry and elemental analysis. High-performance liquid chromatographic (HPLC) analysis of the 2:1 conjugate confirmed high purity of the sample (96.4%) (Supplementary Figure 2).

As we have discussed elsewhere, the unique angle configuration of Fe-O-Fe (146.359 °) in the crystal structure of Fe(Salen) is important in generating its magnetism [6, 12]. Accordingly, we expected that the magnetism of M-PTX would also be dependent on the molecular structure. In order to select the most appropriate conjugate for the present purpose, we conducted density functional theory (DFT) calculations. As shown in Supplementary Table 1, the 2:1 conjugate (M-PTX B) showed an Fe-O-Fe (Goodenough-Kanamori-Anderson) angle of 140.768 °, which corresponds quite closely to the angle in free Fe(Salen) [6]. Supplementary Table 2 and Supplementary Table 3 show the HOMO-LUMO gaps and the binding energy values of the M-PTX conjugates with different coupling ratios. Based on these results, M-PTX B was chosen for further studies due to firstly ease of preparation together with high reproducibility of chemical synthesis, secondly its magnetic strength, and chemical stability. Therefore, hereafter the term M-PTX refers to the 2:1 conjugate M-PTX B unless otherwise noted.

The magnetization of M-PTX NPs was measured with an electron spin resonance (ESR) spectrometer and a superconducting quantum interference device (SQUID) (Quantum Design MPMS7 system) [6]. The ESR spectrum (Figure 1C) was similar to those of Fe(Salen) [8] and Fe(Salen)-conducting copolymer core-shell nano-assemblies [9]. SQUID plots of magnetization versus magnetic field at 37° C (310 K) revealed that M-PTX NPs showed positive magnetization with increasing applied magnetic field (Figure 1D), indicating that the NPs have a suitable magnetization value for magnet-driven drug delivery.

Transmitting electron microscope (TEM) and scanning electron microscope (SEM) (Figure 1E) studies of M-PTX NPs in suspension (50 mM) showed a relatively uniform particle size range of 500∼800 nm. Dynamic light scattering studies showed that M-PTX and PTX NP dispersions have uniform size distributions of ∼551.4 nm (polydispersity index = 0.268) and ∼571 nm (polydispersity index = 0.552), respectively (Supplementary Figure 3). The zeta potentials of M-PTX and PTX were +15.2 mV and –18.5 mV, respectively, indicating fair colloidal stability in aqueous solution. However, the stability gradually decreased during storage (> 15–30 min) due to the inherent hydrophobicity of NPs.

We also performed powder X-ray diffraction (XRD) (Supplementary Figure 4). The peaks of M-PTX NPs were different from those of Fe(Salen) (non-recrystallized form [11], recrystallized form [12]) and PTX [13], indicating that the two drugs had been successfully conjugated, and were not simply present as a physical mixture.

### MR contrast imaging of M-PTX NPs

An *in vitro* sample of M-PTX NPs exhibited a concentration-dependent (0–0.19 mM) negative signal on a T2-weighted image, indicating that M-PTX can be visualized by MRI (Figure 1F). The relaxivity of the M-PTX particles in saline solution was 379.1 s^–1^ mM^–1^ at 7 tesla (at 23° C; calculated based on the molecular weight of M-PTX).

### Cellular uptake of M-PTX NPs

**Figure 2:**
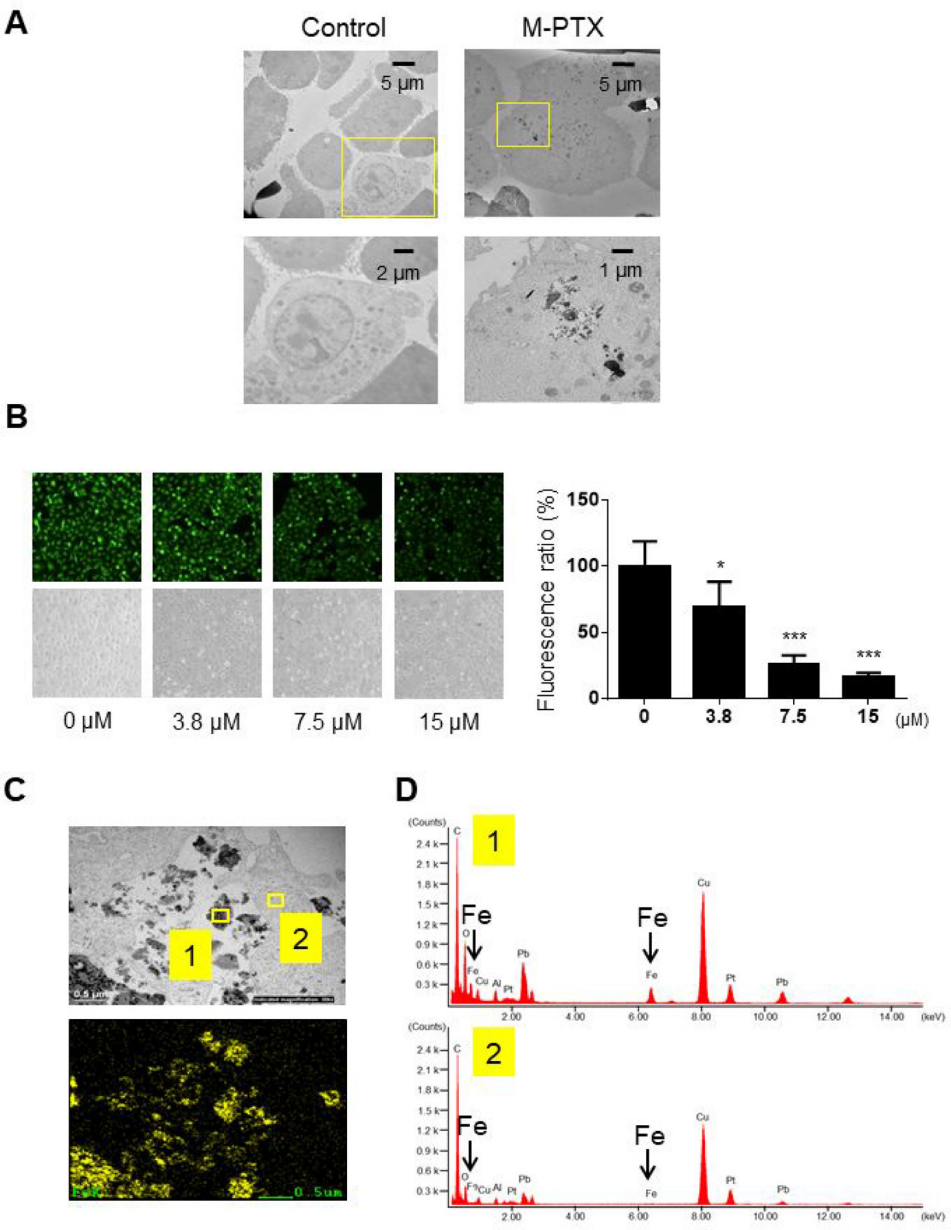
Cellular uptake of M-PTX NPs in human squamous cell carcinoma (**A**) Cellular uptake of M-PTX NPs was analyzed by transmission electron microscopy (TEM) and energy-dispersive X-ray (EDX) spectroscopy in OSC-19 cells. (*left*) TEM of control cells, (*right*) TEM of cells after incubation with 30 μM M-PTX NPs. Calibration bars (5 (*upper*), 2 (*left lower*) and 1 (*right lower*) µm) are shown. Note that cells were shrunken and vacuolated in the presence of M-PTX NPs, indicative of apoptotic cell death. (**B**) Representative images of calcein using a fluorescence microscope and optical microscope. Ratios of calcein fluorescence are shown below (*n =* 4, ^*^p < 0.05, ^***^p < 0.001 vs. control). Note that cellular fluorescence was decreased in the presence of M-PTX. (**C**) M-PTX NPs in a high-power field of TEM. Yellow boxes indicate M-PTX NPs and cytoplasm, which were analyzed by EDX in (D). The calibration bar (500 nm) is shown. (**D**) Spectrum analysis of M-PTX NPs by EDX. Yellow box #1 (M-PTX NPs) in (C) showed the peaks of iron, while yellow box #2 (cytoplasm) did not. Arrows indicate specific peaks of iron.

We confirmed that M-PTX NPs were taken up into human squamous cell carcinoma OSC-19 cells by means of TEM and energy-dispersive X-ray (EDX) analysis. TEM revealed that cells were shrunken and vacuolated, proceeding to apoptotic cellular death, in the presence of M-PTX NPs (Figure 2A). The cellular uptake efficiency of M-PTX NPs was also examined using calcein (CA) as a fluorescent probe for cellular iron [6]. When CA binds to iron, the intensity of the fluorescence decreases [7]. When OSC-19 cells were incubated in the presence of calcein and M-PTX NPs, the cellular fluorescence intensity was decreased in a concentration-dependent manner, suggesting that M-PTX NPs were taken up, at least to some extent, by the cells (Figure 2B). We also performed elemental analysis of M-PTX particles by EDX at the sites indicated by yellow boxes in Figure 2C), and substantial peaks due to iron were seen in the cellular site (Figure 2D) compared with the background site (Figure 2D).

### M-PTX retains the anti-cancer characteristics of PTX

**Figure 3:**
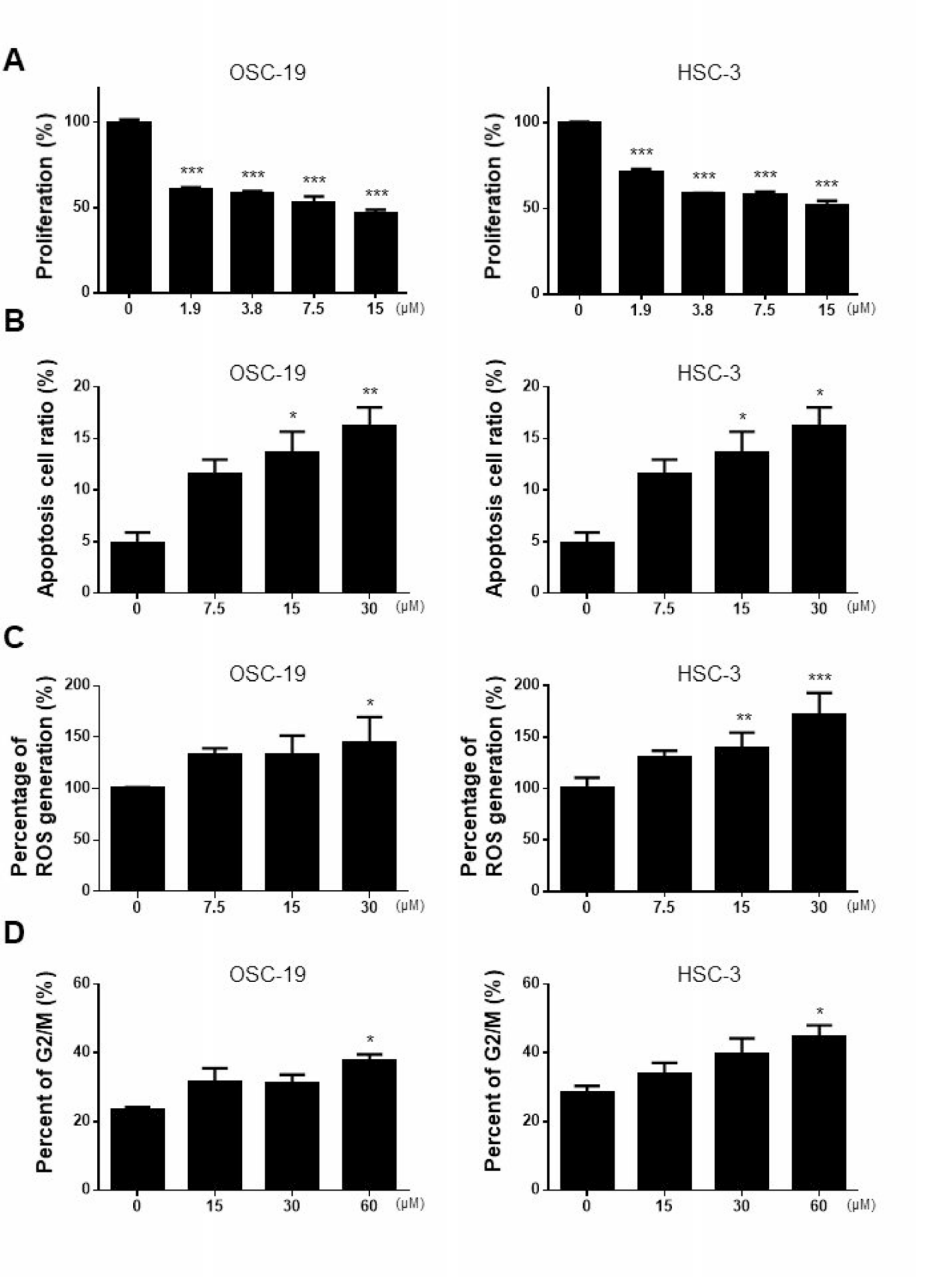
Anti-cancer effect of M-PTX in human squamous cell carcinoma (**A**) Anti-cancer effect of M-PTX. OSC-19 (human squamous cell carcinoma) and HSC-3 (human squamous cell carcinoma) were cultured in the presence of 1.9, 3.8, 7.5 or 15 µM M-PTX. Anti-cancer effect was analyzed by means of XTT assays in comparison with that of intact PTX (*n =* 4, ^***^p < 0.001 vs. control). (**B**) Cell apoptosis rate was assessed by FACS detection. M-PTX induced apoptosis in OSC-19 cells (*n =* 4, ^*^p < 0.05, ^**^p < 0.01) and HSC-3 cells (*n =* 6, ^*^p < 0.05 vs. control) in a dose-dependent manner (0, 7.5, 15 or 30 μM) for 6 hours. (**C**) Effect of M-PTX on ROS production in OSC-19 and HSC-3. M-PTX generated ROS in a concentration-dependent manner for 24 hours (*n =* 4, ^*^p < 0.05, ^**^p < 0.01, ^***^p < 0.001 vs. control). (**D**) Cell cycle analysis. The cell cycles of OSC-19 and HSC-3 cells were analyzed at 6-hours intervals in the presence or absence of M-PTX. M-PTX retained the characteristic anti-cancer mechanism of PTX, *i.e.*, induction of G2/M arrest (*n =* 4, ^*^p < 0.05).

The anti-cancer activities of M-PTX and PTX against OSC-19 and HSC-3 human squamous carcinoma cells were compared by means of XTT assay. M-PTX NPs exhibited a potent, dose-dependent anti-cancer effect on these cells after 24 hours (Figure 3A), and M-PTX and PTX showed similar cytotoxic effects up to 60 hours (Supplementary Figure 5). M-PTX and PTX also showed similar anti-cancer effects against A549 (human alveolar adenocarcinoma) and OVK18 (ovarian cancer cell lines) after 24 hours (Supplementary Figure 6).

PTX induces cell apoptosis [14], and M-PTX NPs similarly showed dose-dependent induction of apoptosis in OSC-19 and HSC-3 cells (Figure 3B). PTX also generates reactive oxygen species (ROS) [15], and M-PTX NPs similarly generated ROS (Figure 3C). Further, PTX stabilizes tubulin polymerization, resulting in arrest at the G2/M phase of the cell cycle [3], and we found that the M-PTX also induces G2/M arrest of OSC-19 and HSC-3 cells (Figure 3D).

**Figure 4:**
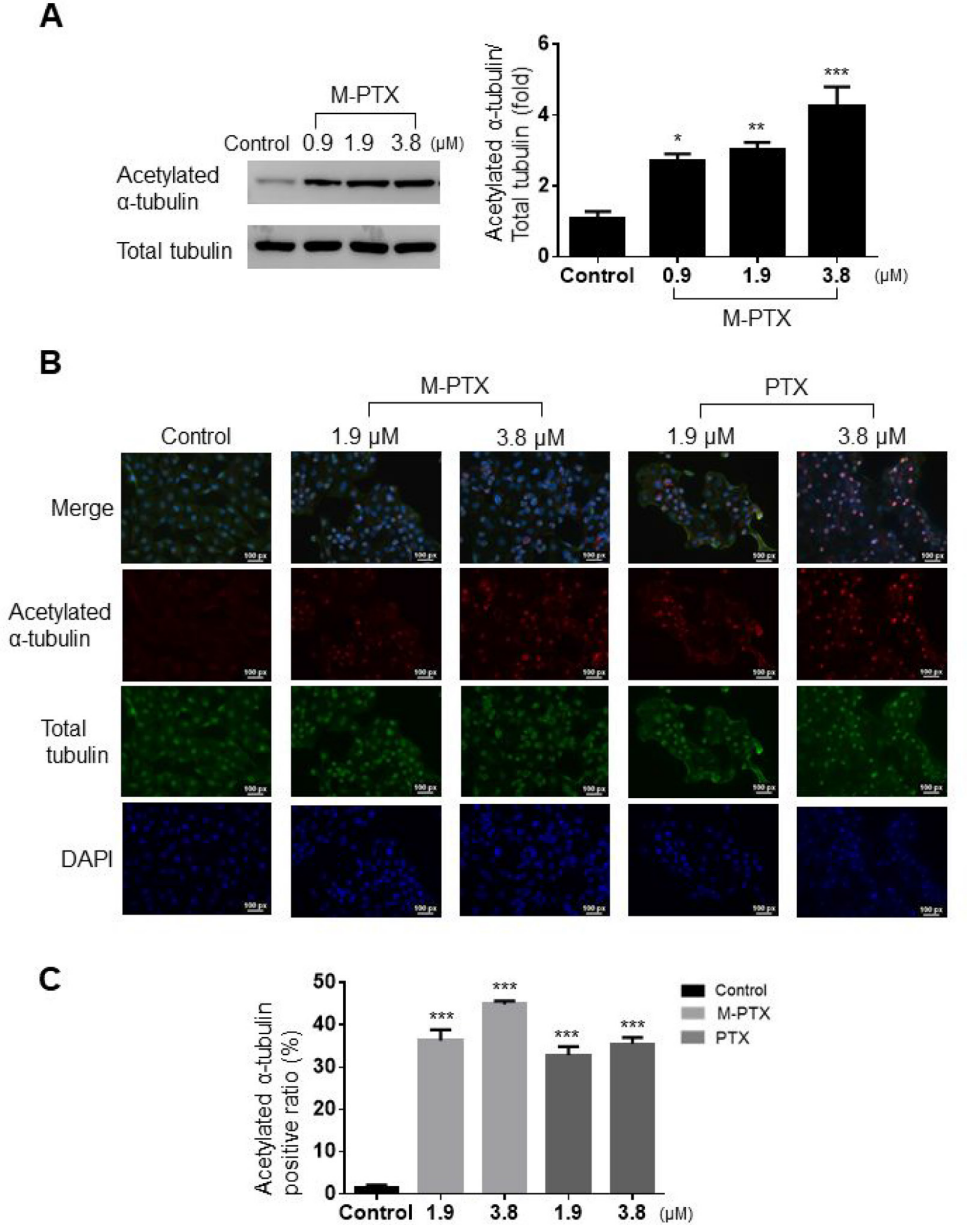
M-PTX NPs inhibited acetylation of α-tubulin and microtubule polymerization in human squamous cell carcinoma (**A**) Western blot analysis of protein expression of acetylated α-tubulin and total α-tubulin. Densitometric analysis (bar graph) of the western blot showed that M-PTX acetylated α-tubulin in a dose-dependent manner in OSC-19 cells (*n =* 4, ^*^p < 0.05, ^**^p < 0.01, ^***^p < 0.001 vs. control). (**B**) Representative images of immunocytochemistry for acetylated α-tubulin (red), α-tubulin (green) and nuclei (blue) in the absence or presence of M-PTX (0, 1.9 or 3.8 μM) or PTX (0, 1.9 or 3.8 μM). (**C**) Ratio of acetylated α-tubulin-positive cells. (*n =* 4, ^***^p < 0.001).

It is known that the C-terminal of PTX acetylates the N-terminal of α-tubulin and prevents depolymerization, thereby disabling the mitotic spindle for cell division [16, 17]. We found that M-PTX NPs inhibited microtubule polymerization in the same manner as did PTX, by means of Western blotting and immunocytochemical analyses. M-PTX NPs also dose-dependently inhibited acetylation of α-tubulin in OSC-19 cells for 24 hours (Figure 4A). This result was also supported by immunocytochemistry (Figure 4B and 4C).

Overall, these results show that conjugation with Fe(Salen) did not impair the activity of PTX against various types of cancer cells, or alter its mechanism.

### Examination of lethal dose and systematic side-effects of M-PTX NPs after injection into mice

We examined the lethal dose and systematic side effects of M-PTX NPs after intravenous injection (0, 100, 150, 200 mg/body) into mice via a tail vein. The maximum non-lethal dose was 100 mg (Supplementary Table 4). Blood chemistry tests (creatinine (Cre), total bilirubin (Tbil), AST, ALT and lactate dehydrogenase (LD) ) revealed no significant change at 7 days after M-PTX injection at 0, 100, or 200 mg/body (Supplementary Table 4).

### The anti-cancer effect of M-PTX was increased by permanent magnet-guided accumulation *in vitro*

**Figure 5:**
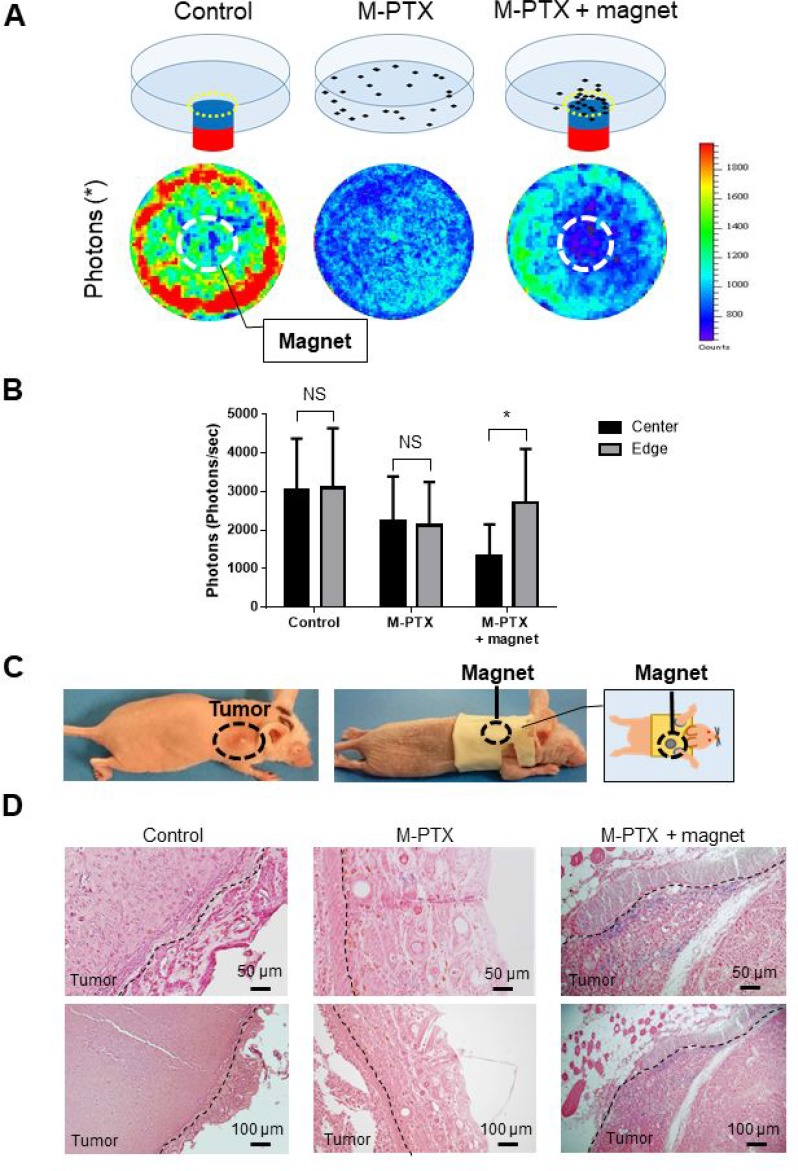
M-PTX is attracted by a permanent magnet in model mice *in vivo* (**A**) Cell viability in the presence of M-PTX with/without magnet. Representative IVIS images in the absence (*left*) or presence of M-PTX without (*center*) / with (*right*) a permanent magnet under the center of the culture dish. (**B**) Plot of bioluminescence signal with IVIS (*n =* 4, control vs. M-PTX vs. M-PTX with magnet). Cell viability was determined in term of luciferase activity by intensity measurement with an IVIS imaging system. Bar graphs show the determination of cell viability with IVIS (*center* and *edge*) (*n =* 4, NS, not significant, ^*^p < 0.05). (**C**) Picture of mouse wearing the jacket carrying a permanent magnet. (**D**) Representative histological pictures of tumor tissues from each group. Berlin blue staining (for M-PTX) day 7 are shown. Blue staining demonstrates accumulation of M-PTX without (*center*)/with (*right*) a permanent magnet or saline (*left;* control). Calibration bars (50 (*upper*) and 100 (*lower*) µm) are shown.

We then examined whether a permanent magnet could accumulate M-PTX NPs at a target site. OSC-19 cells were incubated at 37° C in the absence or presence of M-PTX NPs with/without a permanent magnet placed under the center of the culture dish. M-PTX NPs exhibited anti-cancer activity towards all cells in the culture dish in the absence of the magnet, and there was no marked difference between the center and edge of the dish (Figure 5A and 5B). In the presence of the magnet, the anti-cancer effect at the center of culture dish, above the magnet, was greatly enhanced compared with the edge, indicating the magnet attracted M-PTX NPs, causing their pharmacological action to be concentrated in its vicinity.

We next examined whether a similar effect could be observed *in vivo*. An animal model of human oral cancer was established by transfecting OSC-19 cells with luciferase-encoding lentivirus [7], and the resulting cells were xenografted into the right back of mice. A special jacket containing a magnet was placed on the mice, so that the magnet was located above the tumor (Figure 5C). Tumors were harvested at 7 days after intravenous injection of M-PTX NPs, and iron was stained with Berlin blue (Figure 5D). The blue staining of the tumor site clearly indicated that M-PTX NPs had been accumulated around the magnet. Furthermore, a signal reduction at the tumor site on T2*-weighted MRI was observed in the OSC-19 human carcinoma skin-grafted mice equipped with a magnet after M-PTX administration, but not after PTX administration (Supplementary Figure 7). These results showed that M-PTX was accumulated at the tumor site by the permanent magnet, and this accumulation could be observed by MRI *in vivo*.

### M-PTX suppresses tumor growth in mice similarly to commercial PTX

We set out to compare the anti-cancer effects of M-PTX and PTX *in vivo*, using the same mouse model as in Figure 5D. At 3 days after implantation of OSC-19 cells, mice were segregated into three groups (Supplementary Figure 8). Saline, M-PTX or PTX NPs (12 mg/kg per mouse) was injected intravenously into a tail vein. Tumor size was increased 1253% in the control group after 14 days, whereas the increase was significantly smaller (about one-half) in the M-PTX NPs and PTX NPs-injected groups.

### Magnetically guided focal delivery enhanced the anti-cancer effect of M-PTX *in vivo*

**Figure 6:**
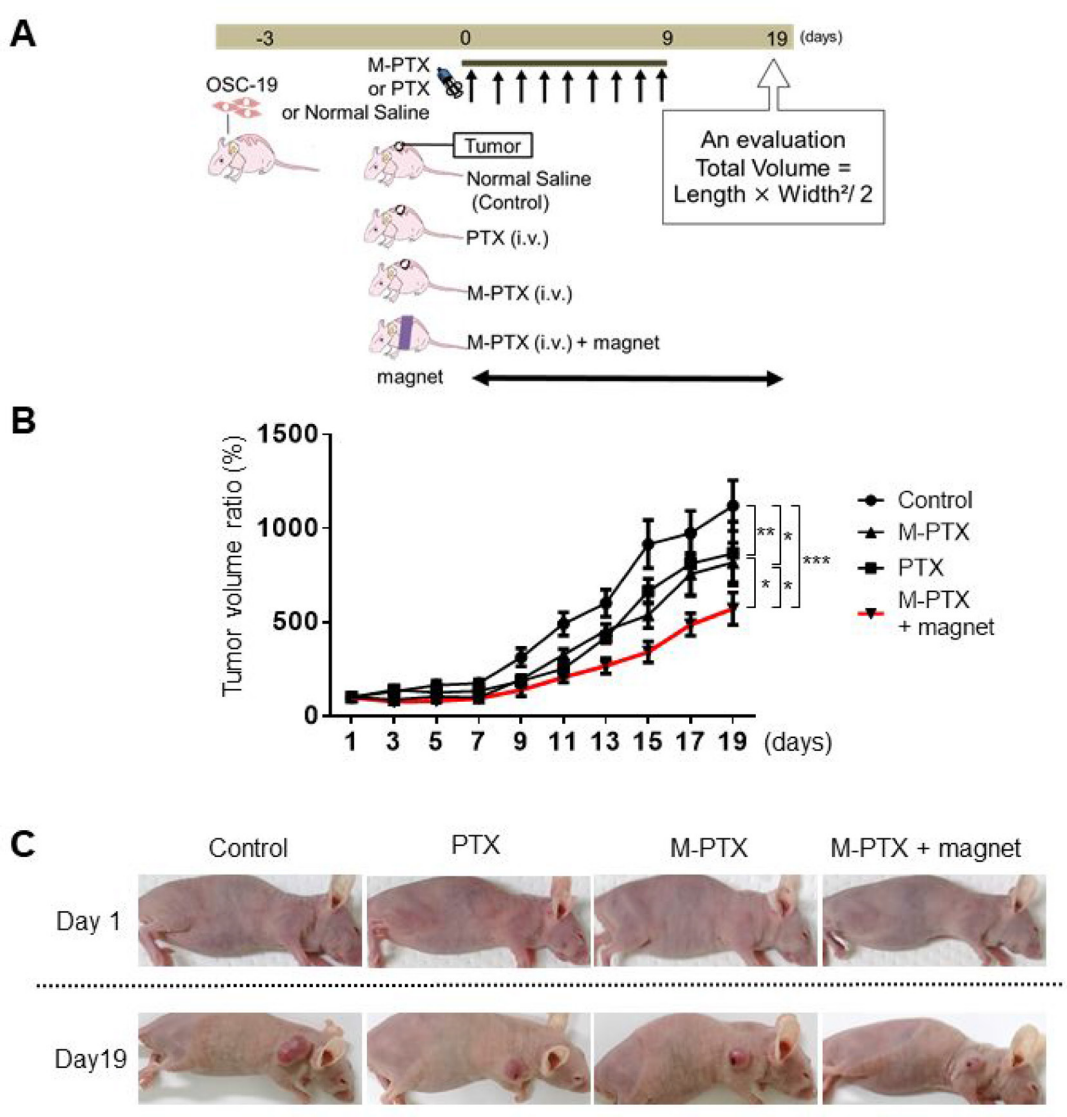
Magnet enhances anti-cancer effect of M-PTX in mice (**A**) Treatment schedule for oral cancer model mouse experiment. (**B**) Regression rate based on manual measurement of tumor volume changes in mouse back (Control (saline), PTX, M-PTX and M-PTX with permanent magnet). The red line indicates the ratio in the M-PTX with permanent magnet group (*n =* 7–8, ^*^p < 0.05, ^**^p < 0.01, ^***^p < 0.001). (**C**) Representative pictures of mouse back tumor in each treatment group. Control (saline), PTX, M-PTX and M-PTX with permanent magnet.

Next, we examined whether magnetically guided delivery of M-PTX NPs had a greater effect compared with the same preparation without the magnet, using the same mouse model as in Figure 5D (dose: 12 mg/kg per mouse). Mice were implanted with OSC-19 cells for 3 days, then segregated into four groups and treated according to the schedule in Figure 6A. None showed neuropathy, focal skin reactions, cytopenias and kidney function when M-PTX or PTX was administered at the current dose in our animal study. The tumor size in the PTX and M-PTX NPs-injected group was significantly reduced compared to the control group at Day 19, while that in the M-PTX NPs-treated group with magnetic guidance was significantly reduced compared to the PTX and M-PTX NPs-treated group without magnetic guidance (Figure 6B and 6C). Thus, magnetic guidance enhanced the anti-cancer effect of M-PTX NPs. Similar results were reproducibly obtained from photon flux measurements with IVIS (Supplementary Figure 9A and 9B). Peripheral neuropathy was not observed in our all study.

## DISCUSSION

Our findings show that magnetically guided delivery of M-PTX NPs with a simple permanent magnet enhanced the anti-cancer effect in a mouse model of oral cancer *in vivo*. This is an important result, because it means that a lower dose can be used for treatment, thereby minimizing systemic side effects. A higher M-PTX concentration could be achieved at the tumor site, where the drug was delivered by magnet, in comparison to the other sites. This is a major advantage over the conventional PTX treatment. We believed that M-PTX is particularly useful for skin, breast, or oral cancer; these tumors are located on the body surface, where a permanent magnet is readily applied. Therefore, M-PTX is a promising anticancer drug for magnetic drug delivery. Further investigation may be necessary to use M-PTX for patients. We need to optimize the usage of a permanent magnet, *i.e.*, magnetic strength, exposure time, its placement, or supporting apparatus for a magnet, in order to achieve the maximal drug delivery effect. A key advantage of the present system is that the magnetically responsive moiety, Fe(Salen), is covalently linked to the well-known anti-cancer agent PTX, affording a stable conjugate. This is in marked contrast to conventional micelle-based drugs, which contain physical mixtures of magnetic particles and anti-cancer drug, and are susceptible to breakdown or loss of potency. We believe the present strategy could be easily extended to other conventional anti-cancer agents, whether or not the Fe(Salen) moiety is employed as one of the partners. For example, we have covalently coupled methotrexate (MTX), pazopanib (Votrient^®^) and rabbit immunoglobulin G (IgG) antibody, aiming to reposition these drugs for new applications [18]. Such integrated drug conjugate systems could revolutionize the treatment of a variety of human cancers.

It should be noted that the magnetic property of Fe(Salen) enables generation of local hyperthermia when exposed to an AMF. Fe(Salen) has intrinsic anti-cancer activity, and we have previously shown that this is dramatically enhanced by the addition of magnetically guided delivery and AMF-induced heating [7]. In other words, combined hyperthermia and chemotherapy with a single drug was an extremely effective strategy. We had hoped to apply this strategy to M-PTX as well, but unfortunately M-PTX NPs failed to generate substantial heat when exposed to AMF, presumably due to the antiferromagnetic coupling of M-PTX NPs at low crystallinity. However, as magnetism is related to the Fe-O-Fe angle (Supplementary Table 1), it may be possible to synthesize an M-PTX derivative suitable for generating hyperthermia. Further experiments along this line are in progress.

In order to address issues such as poor water-solubility, difficult colloidal processability, and potential cytotoxicity to healthy cells, we recently designed Fe(Salen)-loaded nanocarriers as an advanced formulation platform for delivery of insoluble drugs [9]. Such nanocarriers might also be useful to deliver conjugates of conventional anti-cancer drugs with Fe(Salen). We believe this represents a promising strategy for synergistic drug development to maximize chemotherapeutic efficacy and minimize toxicity in collaboration with disciplinary fields by designing and improving computational approach and systematic chemical synthesis in collaboration with engineers and scientists.

## MATERIALS AND METHODS

### Reagents

*N,N’-Bis(salicylidene)ethylenediamine* was purchased from Sigma-Aldrich (Missouri, USA). Fe(Salen) was purchased as *N*,*N*’-bis(salicylidene)ethylenediamine iron(II) from Tokyo Chemical Industry Co., Ltd. (Tokyo, Japan) and used as received or after sonication. As the drug is poorly soluble, suspensions were prepared by extensive sonication for 6 hours in normal saline and in very low concentrations (0.5%) of ethanol and Cremophor (Wako Pure Chemical Industries, Osaka, Japan) for cellular assays and animal studies. Paclitaxel (PTX) was purchased from Wako Pure Chemical Industries (Osaka, Japan).

### Covalent linking of PTX to Fe(Salen)

*tert*-Butyl (3-formyl-4-hydroxyphenyl) carbamate (5) [19] and 2’-*O*-4-nitrophenoxycarbonyl-PTX (8) were prepared according to the literature [20].


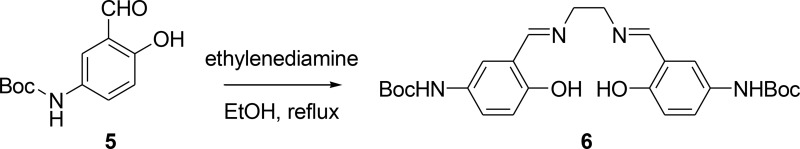


### *N,N’*-Bis(5-*tert*-butoxycarbonylamino-2-hydroxybenzylidene)ethylenediamine (6)

A solution of 5 (260 mg, 1.1 mmol) in anhydrous EtOH (10 mL) was heated to reflux and then ethylenediamine (33 mg, 0.55 mmol) in anhydrous EtOH (10 mL) was added dropwise to the hot solution. After the addition, the mixture was stirred at reflux for 0.5 h. The precipitate was collected by filtration, washed with EtOH (50 mL), and dried in vacuo to give 200 mg of Schiff base ligand 6 as pale yellow needles. ^1^H NMR (300 MHz, DMSO-*d*_6_): δ 1.46 (s, 18H), 3.90 (s, 4H), 6.77 (d, *J* = 8.7 Hz, 2H), 7.28 (dd, *J* = 8.7, 2.1 Hz, 2H), 7.57 (s, 2H), 8.54 (s, 2H), 9.21 (s, 2H) 12.97 (s, 2H).


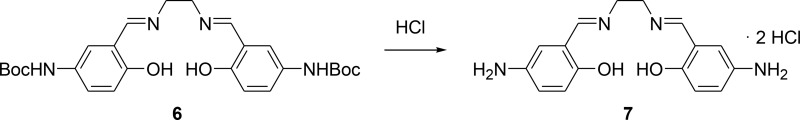


### *N,N’*-Bis(5-amino-2-hydroxybenzylidene)ethylenediamine dihydrochloride (7)

To a solution of 6 (200 mg, 0.4 mmol) in anhydrous dichloromethane (200 mL) was added dropwise a solution of 4 M HCl in ether (2 mL). The solution was stirred at room temperature for 3 h, then filtered, and the precipitate was washed with dichloromethane and ether to afford **7**. ^1^H NMR (300 MHz, DMSO-*d*_6_ + D_2_O): δ 3.04 (s, 4H), 4.14 (br s, 6H), 7.07 (d, *J* = 9.0 Hz, 2H), 7.41 (d, *J* = 9.0 Hz, 2H), 7.58 (s, 2H), 8.90 (s, 2H), 10.16 (s, 2H).


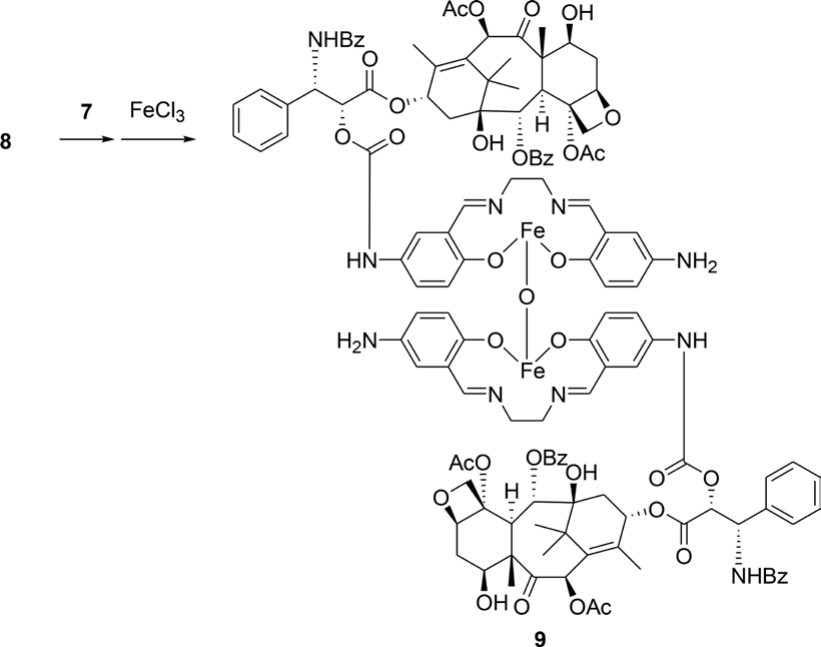


### Compound (9)

To a solution of 8 (700 mg, 0.687 mmol) and K_2_CO_3_ (285 mg, 2.06 mmol) in anhydrous *N*,*N*-dimethylformamide (DMF) (60 mL) was added dropwise a solution of **7** (520 mg, 1.40 mmol) in anhydrous DMF (60 mL) under nitrogen at –30° C. The mixture was stirred at –20° C for 3 h, and filtered. The filtrate was concentrated in vacuo to give a crude product. This was suspended in MeOH (100 mL), and triethylamine (209 mg, 2.06 mmol) and FeCl_3_·6H_2_O (223 mg, 0.824 mmol) was added to the solution. The resulting dark brown mixture was stirred at 40° C under nitrogen for 30 min, then evaporated in vacuo to give a crude solid, which was recrystallized from MeOH and diethyl ether to afford the desired compound 9 (537 mg, 63% yield) as a brown solid. HPLC (>96.7% purity). MS (API-ES) (*m*/*z*): [M+2H]^+^ Calcd for C_128_H_132_Fe_2_N_10_O_35_, 2481.76; found, 2481.6.

### Magnet

A flat, square magnet (383 mT surface magnetic flux density, neodymium-based magnet of grade NF48H, 5* 5* 3 mm size) was purchased from Sagami Chemical Metal Co., Ltd. (Tokyo, Japan) (Figure 6).

### Electron spin resonance spectroscopy

Electron spin resonance (ESR) measurement was performed using a Bruker BioSpin EMXPLUS 8/2.7S spectrometer system (Bruker BioSpin, Massachusetts, USA), operating at 9.860 GHz modulation frequency at room temperature [7]. Microwave power and modulation amplitude were 1 mW and 1 G, respectively. Powder samples were prepared in a glass capillary sample holder and placed in a probe head.

### Superconducting quantum interference device (SQUID)

The magnetization of M-PTX was measured with a superconducting quantum interference device (SQUID) (Quantum Design MPMS7 system, Quantum Design, Inc., California, USA) [7]. Each sample was enclosed in a plastic capsule (Quantum Design) for measurement. Plots of magnetization versus magnetic field at 37° C (310 K) revealed that the M-PTX exhibited paramagnetic behavior. Magnetization (M (emu/g)) with an applied magnetic field (H/Oe) was examined in the range from −1,000 to 1,000 Oe.

### Transmitting electron microscope (TEM) and scanning electron microscope (SEM) images of M-PTX NPs

The morphology and size distribution of the M-PTX NPs were observed using a transmitting electron microscope; TEM (H-7500, Hitachi, Ltd., Tokyo, Japan) and a scanning electron microscope; SEM (S-4800, Hitachi).

### MR imaging

MR image acquisition was performed with a 7.0 Tesla magnet (BioSpec Avance-III, Bruker-Biospin, Massachusetts, USA) an 85-mm diameter volume resonator for transmission (Bruker-Biospin), and a 35-mm diameter 8-ch phased array coil (T20034V3, Bruker-Biospin) for reception. The sample temperature was maintained at 23° C using a gradient-coil cooling system and air conditioner. Two-dimensional multi-spin-echo images were acquired with the following parameters: repetition time (TR) / echo time (TE) = 3,000/10–100 ms in steps of 10 ms (10 echoes); field of view (FOV) = 38.4 × 38.4 mm^2^; matrix = 256 × 256; resolution = 150 × 150 μm; number of slices = 1; slice thickness = 2.0 mm; slice direction = horizontal; and number of acquisitions = 1. The scanning time was 12 min 48 sec. After image acquisition, the T_2_ and R_2_ were estimated using the MRVision software (version 1.6.8, MR Vision Co., Massachusetts, USA). The transverse relaxivity (r_2_) was calculated according to the equation; r_2_ = (R_2obs_ -R_2d_) / [M-PTX] (R_2obs_; R_2_ of the sample, R_2d_; R_2_ of the aqueous solution, [M-PTX]; M-PTX concentration).

### Cell lines and cell culture

Cells were cultured in an incubator at 37° C in an atmosphere of 5% CO_2_ in air. Human oral squamous cell carcinoma cell lines OSC-19 and HSC-3 were purchased from the Health Science Research Resources Bank (Japan Health Sciences Foundation, Tokyo, Japan). OSC-19 cells that had been transfected with luciferase-encoding vector were a gift from Dr. Kioi. In all cases, early passage cultures were stored and used for experiments. These cell lines were cultured in Dulbecco’s modified Eagle’s medium (DMEM (High Glucose), Sigma-Aldrich, Missouri, USA) with L-glutamine, phenol red and sodium pyruvate medium containing 10% fetal bovine serum (FBS), 1% penicillin-streptomycin and 1% L-glutamine.

### Scanning transmission electron microscope and elemental analysis

Cellular uptake of M-PTX NPs in OSC-19 cells was analyzed by scanning transmission electron microscopy (STEM, Hitachi High Technologies HD-2700, Hitachi High Technologies Corporation., Tokyo, Japan) [6]. Bright-field (BF) and annular dark-field (ADF) TEM images were obtained to detect M-PTX NPs taken into OSC-19 cells. Energy dispersive X-ray spectroscopy (EDX) and X-ray mapping were used to analyze the composition of the particles. All TEM images, spectra and mapping images were taken at an accelerating voltage of 200 kV.

### Calcein assay

Calcein-AM was purchased from Sigma-Aldrich (Missouri, USA) [6]. OSC-19 cells in medium were seeded into the wells of two 24-well plates and incubated for 24 hours. DMEM Hi-glucose medium was changed to serum-free medium, followed by addition of 1 μM calcein and incubation at 37° C for 1 hour. Then M-PTX NPs (0, 3.8, 7.5 and 15 μM) were added at room temperature in darkness. The cells were further incubated at room temperature for 3 hours. Cellular uptake of M-PTX NPs was analyzed by fluorescence microscopy.

### Cell proliferation assay

Cell proliferation assay was performed using a commercial kit, *Sodium 2,3,-bis(2-methoxy-4-nitro-5-sulfophenyl)-5-[(phenylamino)-carbonyl]-2H-tetrazolium inner salt* (XTT) Cell Proliferation Assay Kit (ATCC, Virginia, USA), as previously reported [6–8]. Briefly, cells were seeded into the wells of a 96-well plate. The inoculated plate was incubated at 37° C for 2 hours in a humidified atmosphere of 5% CO_2_ in air. Blank control wells contained medium without M-PTX. M-PTX was added to the other wells, and the plate was incubated for 24 hours under the same conditions. Activated-XTT Solution (50 μl) was added to each well, and the plate was returned the incubator for 2 hours. The absorbance of all wells was measured with a micro plate reader.

### Apoptosis assay

Apoptosis assay was performed as previously described [7, 21]. OSC-19 and HSC-3 cells were seeded on 6 cm dishes (1.0 × 10^5^ cells per dish), and incubated for 24 hours. M-PTX NPs were then added to give 7.5, 15 or 30 μM, and incubation was continued for 6 hours. Cells were washed twice with cold PBS, and transferred into culture tubes. APC Annexin V and 7-AAD (BD Biosciences, California, USA) were added to the tubes, and incubation was continued for 15 min at RT (25° C) in darkness, followed by measurement with a FACS Canto™ II (Japan Becton, Dickinson and Company, Tokyo, Japan) within 1 hour.

### Measurement of reactive oxygen species (ROS) assays

Measurement of ROS was performed as previously described [3, 18]. OSC-19 and HSC-3 cells were incubated for 24 hours in 96-well plates (5.0 × 10^3^ cells per well). M-PTX NPs were then added to give 7.5, 15 or 30 μM, and incubation was continued for 24 hours. Intracellular ROS was measured using a fluorescent dye, 2′,7-dichlorofluorescein diacetate (DCFH-DA; Sigma Aldrich, Missouri, USA). ROS production was measured using a microplate reader equipped with a spectrofluorometer (ARVO-Mx, PerkinElmer, Massachusetts, USA) at an emission wavelength of 538 nm and an excitation wavelength of 485 nm.

### Cell cycle analysis

Cell cycle analysis was performed using The Cycletest™ Plus DNA Reagent Kit (BD Biosciences, California, USA) [15]. OSC-19 and HSC-3 cells were seeded on 6 cm dishes, and incubated for 24 hours. M-PTX NPs were then added to give 15, 30 or 60 μM, and incubation was continued for 6 hours. Briefly, OSC-19 and HSC-3 cells were washed in PBS and fixed in 90% ethanol. Fixed cells were then washed twice in PBS, stained with 50 μM propidium iodide containing 5 μg/ml DNase-free RNase for 1 hour, and then analyzed by flow cytometry using a FACS Canto™ II (Japan Becton, Dickinson and Company, Tokyo, Japan).

### Western blot analysis

Western blot analyses were performed as described [22]. Immunoblotting for α-tubulin and acetylated α-tubulin were performed with antibodies from Abcam (Cambridge, UK) and Sigma-Aldrich (Missouri, USA), respectively. Signal intensities of the bands were quantified with Image J software (NIH).

### Immunocytochemistry

Cells were seeded and cultured in the presence of M-PTX or PTX. After 24 hours, cells were stained with α-tubulin antibody (Abcam, Cambridge, UK) and acetylated α-tubulin (Sigma Aldrich, Missouri, USA), and observed with a fluorescence microscope [7, 17].

### Evaluation with IVIS of *in vitro* anti-cancer activity towards human oral cancer cells of M-PTX guided with a permanent magnet

Analysis using an *in vivo* imaging system (IVIS, Xenogen, California, USA) was performed as reported [7, 8]. OSC-19 cells were seeded on 6 cm dishes (5.0 × 10^4^ cells per dish) and incubated for 24 hours. Then, M-PTX was added, and the cells were incubated at 37° C for 24 hours with or without a permanent magnet (2 cm in diameter). D-Luciferin (4.7 mg/well) was added, and after 15 minutes, the bioluminescence signal was examined with IVIS.

### Evaluation of M-PTX accumulation in a mouse model implanted with human oral cancer, using a permanent magnet for drug delivery

OSC-19 cells that had been transfected with luciferase-encoding vector were implanted into the back of Balb/c Slc-nu/nu mice (4–5 weeks old; 4 mice/group) (SLC, Shizuoka, Japan) to create a human oral cancer model. The tumors were allowed to grow to a size of 3–5 mm, and then M-PTX NPs (12 mg/kg per mouse) were injected into a tail vein for 7 days repeatedly. A square magnet (383 mT) was used to generate the magnetic field for drug delivery. It was placed in a special jacket that kept it in contact with the top of the tumor mass. The tumors were harvested after 7 days of intravenous injection of M-PTX NPs, and stained with Berlin blue, which colors blue with iron. The changes in skin, fur, eyes and mucous membranes, central nervous systems, respiratory, circulatory, autonomic, and somatomotor activity and behavioral patterns were observed [23]. Mechanical allodynia was assessed by the von Frey test [24, 25].

### Intravenous injection of PTX or M-PTX NPs in a mouse model implanted with human oral cancer

Saline or PTX NPs or M-PTX NPs (12 mg/kg per mouse) were injected into a tail vein of the model mice daily for 9 days. A square magnet (383 mT) was used to generate the magnetic field for drug guidance (M-PTX + magnet group). It was placed in a special jacket that kept it in contact with the top of the tumor mass. The tumor volume ratio was calculated by dividing the volume of each tumor by the baseline volume every other day. The size of the tumors was measured under general anesthesia daily for 19 days. Tumor volume was determined using the following formula:

$$\mathrm{Tumor}\;\mathrm{volume}=0.5\times \left(\mathrm{length}\times {\mathrm{width}}^{2}\right)$$

The changes in skin, fur, eyes and mucous membranes, central nervous systems, respiratory, circulatory, autonomic, and somatomotor activity and behavioral patterns were also observed [23]. Mechanical allodynia was assessed by the von Frey test [24, 25].

### Ethics statement

Animal experiments were performed according to the Yokohama City University guidelines for experimental animals. The Animal Care and Use Committee at Yokohama City University, School of Medicine, approved all animal studies. All experimental protocols were approved by the Animal Care and Use Committee at Yokohama City University, School of Medicine.

### Data analysis and statistics

Statistical comparisons among groups were performed using one-factor analysis of variance (ANOVA). Two-way ANOVA was used to examine in Figure 5A and in the animal study in Figure 6. Statistical significance was set at the 0.05 level.

## SUPPLEMENTARY MATERIALS FIGURES, TABLES AND VIDEO




