# Supplementary material for: Treatment of oral cancer using magnetized paclitaxel
Source: Oncotarget. 2018 Feb 26;9(21):15591–605. doi: 10.18632/oncotarget.24570 (PMC5884650; doi:10.18632/oncotarget.24570)
Supplement: Supplementary file 1 [file oncotarget-09-15591-s001.pdf]

Treatment of oral cancer using magnetized paclitaxel

SUPPLEMENTARY MATERIALS

MATERIALS AND METHODS

Density functional theory (DFT) calculations

The stability and magnetic strength of M-PTX were analyzed by means of first-principles calculations for structures in which one to four molecules of PTX were covalently bound to Fe(Salen) (Supplementary Figure 1). Density functional theory (DFT) calculations were conducted with the DMol3 module in Materials Studio (MS) software (version 8.0, from BIOVIA Inc.) [1] using the generalized gradient approximation (GGA) functional of Becke exchange plus Lee-Yang-Parr correlation (BLYP) [2, 3] in conjunction with the double numerical with polarization (DNP) basis set. All structures were optimized by the natural internal coordinated method [4] together with parallel calculations [5] and considering relativistic effects [6]. Supplementary Table 1 shows the Goodenough-Kanamori-Anderson angle (GKA angle), which is the origin of the magnetism of M-PTX; ferromagnetic character is associated with a GKA angle of 90° and antiferromagnetic character with a GKA angle of 180° [7]. The strength of ferromagnetism increased in the order of M-PTX A, M-PTX C, M-PTX B. However, M-PTX D was almost antiferromagnetic.

Synthesis and characterization of M-PTX

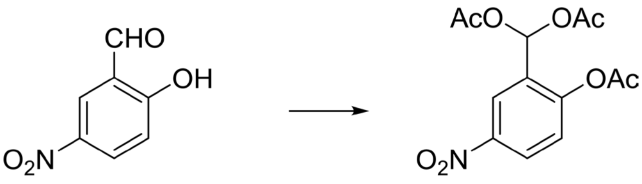

1 **2**

A solution of compound 1 (20 g, 120 mmol), acetic anhydride (240 mL), and H_2SO_4 (24 drops) was stirred at room temperature for 1 h. The solution obtained was poured into ice cold water, and stirred for 0.5 h to hydrolyze the anhydride. Thus formed white solid was collected by filtration, air dried, and recrystallized with EtOAc/hexane to give 35 g of compound 2 (112 mmol, 94% yield) as light brown crystals. ^1H NMR (300 MHz, $CDCl_3$): δ 2.15 (s, 6H), 2.40 (s, 3H), 7.36 (d, J = 9.0 Hz, 1H), 7.95 (s, 1H), 8.31 (dd, J = 2.7, 9.0 Hz, 1H), 8.56 (d, J = 2.7 Hz, 1H).

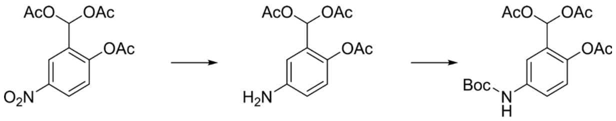

2 **3** **4**

A mixture of compound 2 (46 g, 148 mmol) and 10% palladium on carbon (13.6 g) in THF (350 mL) was hydrogenated with H_2 for 20 h. The reaction mixture was filtered, and the filtrate was used for next step directly. To the filtrate was added TEA (60 g, 594 mmol) portionwise. Then $(Boc)_2O$ (64 g, 296 mmol) was added dropwise to the solution under nitrogen protection. The reaction mixture was stirred at room temperature for about 20 h. The mixture was evaporated to dryness, and the residue was purified on silica gel column (hexane/EtOAc, 2:1). Then the product was recrystallized from hexane/EtOAc to afford the white solid 4 (30.4 g, 79.7 mmol, 54% yield). ^1H NMR (300 MHz, $CDCl_3$): δ 1.52 (s, 9H), 2.10 (s, 6H), 2.32 (s, 6H), 6.54 (s, 1H), 7.04 (d, J = 8.5 Hz, 1H), 7.42 (d, J = 8.5 Hz, 1H), 7.66 (s, 1H), 7.84 (s, 1H).

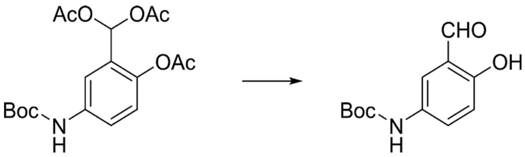

4 **5**

Compound 4 (26 g, 68.2 mmol) was dissolved in MeOH (250 mL), and then NaOH (14 g, 350 mmol) dissolved in water (50 mL) was added. The resulting solution was refluxed for 18 h. After cooling, the solution was neutralized, and the yellow solid was collected by filtration, washed with water, and dried in vacuo to afford the light yellow solid 5 (15.5 g, 65.3 mmol, 96% yield). ^1H NMR (300 MHz, $CDCl_3$): δ 1.53 (s, 9H), 6.44 (s, 1H), 6.93 (d, J = 9.0 Hz, 1H), 7.26-7.30 (m, 1H), 7.87 (s, 1H), 9.88 (s, 1H), 10.79 (s, 1H).

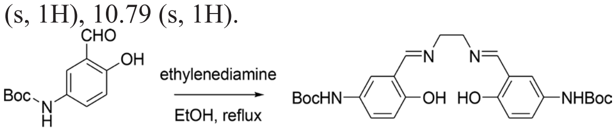

5 **6**

N,N'-Bis(5-*tert*-butoxycarbonylamino-2-hydroxybenzylidene)ethylenediamine (6)

A solution of 5 (260 mg, 1.1 mmol) in anhydrous EtOH (10 mL) was heated to reflux and then ethylenediamine (33 mg, 0.55 mmol) in anhydrous EtOH (10 mL) was added dropwise to the hot solution. After the addition, the mixture was stirred at reflux for 0.5 h. The precipitate was collected by filtration, washed with EtOH (50 mL), and dried in vacuo to give 200 mg of Schiff base ligand 6 as pale yellow needles. ^{1}H NMR (300 MHz, DMSO-d_6): δ 1.46 (s, 18H), 3.90 (s, 4H), 6.77 (d, *J* = 8.7 Hz, 2H), 7.28 (dd, *J* = 8.7, 2.1 Hz, 2H), 7.57 (s, 2H), 8.54 (s, 2H), 9.21 (s, 2H) 12.97 (s, 2H).

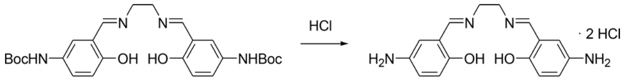

6 7

N,N'-Bis(5-amino-2-hydroxybenzylidene) ethylenediamine dihydrochloride (7)

To a solution of 6 (200 mg, 0.4 mmol) in anhydrous dichloromethane (200 mL) was added dropwise a solution of 4 M HCl in ether (2 mL). The solution was stirred at room temperature for 3 h, then filtered, and the precipitate was washed with dichloromethane and ether to afford 7. ^{1}H NMR (300 MHz, DMSO-d_6 + D_2O): δ 3.04 (s, 4H), 4.14 (br s, 6H), 7.07 (d, *J* = 9.0 Hz, 2H), 7.41 (d, *J* = 9.0 Hz, 2H), 7.58 (s, 2H), 8.90 (s, 2H), 10.16 (s, 2H).

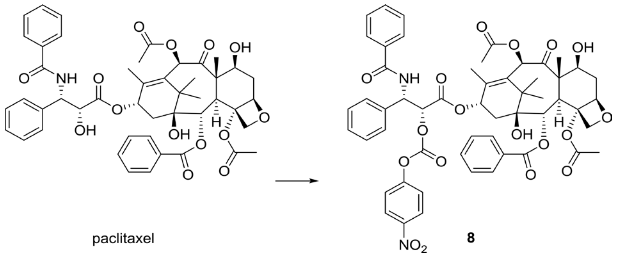

paclitaxel 8

To a solution of Paclitaxel (7 g, 8.2 mmol), DMAP (0.7 g, 5.7 mmol) and Et_3N (3.5 mL, 24.6 mmol) in CH_2Cl_2 (700 mL) at −50° C was added dropwise a solution of 4-nitrophenyl chloroformate (8.2 g, 41.0 mmol) in CH_2Cl_2 under nitrogen. The solution was stirred at −50° C for 3 h, washed with $KHSO_4$ (0.5 N) and brine, and dried over Na_2SO_4. After the volatile compounds were evaporated, the residue was purified by flash chromatography on silica gel (hexane/EtOAc, 2:3) to afford compound 8 (7 g, 6.87 mmol, 84% yield). ^{1}H NMR (300 MHz, $CDCl_3$): δ 1.15 (s, 3H), 1.26 (s, 3H), 1.69 (s, 3H), 1.93 (s, 3H), 2.23 (s, 3H), 2.50 (s, 3H), 2.55 (m, 1H), 3.82 (d, *J* = 7.0 Hz, 1H), 4.21 (d, *J* = 8.4 Hz, 1H), 4.34 (d, *J* = 8.4 Hz, 1H), 4.42 (m, 1H), 4.98 (bd, *J* = 7.7 Hz, 1H), 5.53 (d, *J* = 2.7 Hz, 1H), 5.70 (d, *J* = 7.1 Hz, 1H), 6.10 (bs, 1H), 6.29 (s, 1H), 6.34 (m, 1H), 6.90 (d, *J* = 9.4 Hz, 1H), 7.35 (d, *J* = 9.1 Hz, 2H), 7.37–7.65 (m, 11H), 7.76 (d, *J* = 7.2 Hz, 2H), 8.15 (d, *J* = 7.2 Hz, 2H), 8.26 (d, *J* = 9.1 Hz, 2H).

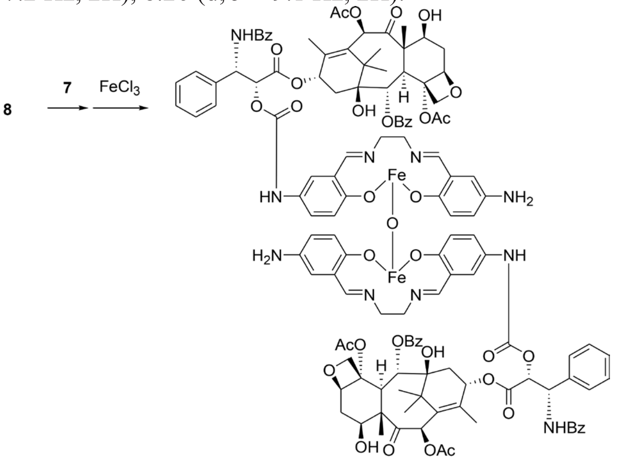

9

Compound (9): To a solution of 8 (700 mg, 0.687 mmol) and K_2CO_3 (285 mg, 2.06 mmol) in anhydrous *N*,*N*-dimethylformamide (DMF) (60 mL) was added dropwise a solution of 7 (520 mg, 1.40 mmol) in anhydrous DMF (60 mL) under nitrogen at –30° C. The mixture was stirred at –20° C for 3 h, and filtered. The filtrate was concentrated in vacuo to give a crude product. This was suspended in MeOH (100 mL), and triethylamine (209 mg, 2.06 mmol) and $FeCl_3 \cdot 6H_2O$ (223 mg, 0.824 mmol) was added to the mixture. The resulting dark brown mixture was stirred at 40° C under nitrogen for 30 min, then evaporated in vacuo to give a crude solid, which was recrystallized from MeOH and diethyl ether to afford the desired compound 9 (537 mg, 63% yield) as a brown solid. HPLC (>96.7% purity). MS (API-ES) (*m/z*): $[M+2H]^+$ Calcd for $C_{128}H_{132}Fe_2N_{10}O_{35}$, 2481.76; found, 2481.6.

Dynamic light scattering (DLS)

The hydrodynamic size and colloidal stability of the M-PTX NPs were determined by dynamic light scattering (DLS) and zeta potential analysis using a Zetasizer Nano ZSP (Malvern Instruments, Ltd., Malvern, UK). Both DLS and zeta potential data were obtained from twelve runs per measurement and exhibited a uniform distribution between runs, which is a criterion of high quality.

Powder X-ray diffraction (XRD) characterization of M-PTX

To prepare a pure M-PTX sample for XRD analysis, 20 mg of M-PTX powder was dissolved in 50 mL of methanol. The solution was stirred thoroughly, then sonicated, and purified by magnetic separation to ensure removal of any undissolved sediment. The resulting clear brown solution was filtered and the filtrate was evaporated *in vacuo* at room temperature. The XRD measurements were performed with a Rigaku Smart Lab horizontal X-ray

diffraction apparatus with a Cu target (output: 45 kV-200 mA). Fe(Salen) powder (TCI, Tokyo, Japan) was used as a standard for XRD.

Real-time cell growth assay

In vitro cell proliferation was measured using a xCELLigence (ACEA Biosciences, California, USA) real-time cellular analysis system [8]. Briefly, the background impedance was measured following the addition of 100 µL of growth medium to 16-well E-plates. Cell suspension containing 5.0×10^3 OSC-19 or HSC-3 cells was seeded into the wells. Attachment and proliferation were monitored using the xCELLigence system. When cells reached the logarithmic growth phase, they were further treated with M-PTX or PTX and continuously monitored for ~100 hours. Impedance change, expressed as cell index (CI), was automatically calculated as live cells interacted with the electrodes in the E-plates, as a parameter of viability or cytotoxicity. Growth curves were normalized to the CI at 48 hours before the anti-cancer effects of M-PTX and PTX became apparent.

In vivo MR imaging of M-PTX or PTX after administration

Nine female BALB/c nude mice (Japan SLC, Shizuoka, Japan) were used for all *in vivo* studies; for evaluation of the accumulation of M-PTX in tumor, five received M-PTX and four were used as the PTX control. The mice were maintained in accordance with the guidelines of the National Institute of Radiological Sciences (NIRS, QST), and all experiments were reviewed and approved by the institute's committee for care and use of laboratory animals.

OSC-19 human carcinoma cells were obtained from the RIKEN BioResource Center (Tukuba, Japan). The cells were maintained in Dulbecco's modified Eagle's medium (D5796, Sigma-Aldrich, Missouri, USA) supplemented with 10 % fetal bovine serum, and incubated in a humidified atmosphere of 5 % CO_2 in air at 37° C. After suspension in phosphate-buffered saline, the cells were subcutaneously grafted (1.0×10^6 cells/50 µl) into the femur area of the mice. The tumors were allowed to grow to 5 mm diameter (Day 0) after tumor cell transplantation.

To evaluate M-PTX accumulation in tumors *in vivo*, M-PTX or PTX was administered three times, once per day, at Days 1, 2 and 3. A permanent magnet was attached at the tumor site for all 3 days. M-PTX or PTX was intravenously administered from a tail vein (80 mg/kg). During Days 0 to 3, there was no significant difference in body weight between M-PTX ($n = 5$) and PTX ($n = 4$) administered mice ($p > 0.05$, 2-way ANOVA with Bonferroni post-hoc tests).

On Day 4 (3 days after the initial administration of the M-PTX or PTX), 2D spin-echo T1-weighted, 2D multi spin-echo T2-weighted and gradient-echo T2*-weighted images were acquired. During the *in vivo* MRI experiments, the rectal temperature of the mice was monitored with an optical fiber thermometer (FOT-L, FISO Technology, Quebec, CA) and maintained at approximately 36.5 ± 0.5° C by warm air flow provided by a homemade automatic heating system based on an electric temperature controller (E5CN, Omron, Kyoto, Japan). The mice were anesthetized with 1.5–2.0% isoflurane (Escain, Mylan, Tokyo, Japan) gas in a 1:5 O_2:room-air mixture.

T1-weighted images were acquired using a multi-spin-echo sequence with the following parameters: TR/TE = 350/9.4 ms; FOV = 30.0×30.0 mm^2; matrix = 200 × 200; resolution = 150 × 150 µm; number of slices = 5; slice thickness = 1.0 mm; slice gap = 0.45 mm; slice direction = transaxial; and NEX = 8. The scanning time was 9 min 20 sec.

T2-weighted images were acquired using a rapid acquisition with relaxation enhancement (RARE) sequence with the following parameters: TR/TE = 3,000/20.3 - 100 ms in steps of 10 ms (10 echoes); FOV = 30.0×30.0 mm^2; matrix = 200 × 200; resolution = 150 × 150 µm; number of slices = 5; slice thickness = 1.0 mm; slice gap = 0.45 mm; slice direction = transaxial; and NEX = 2. The scanning time was 3 min 42 sec.

T2*-weighted images were acquired using a 3D gradient echo sequence (FLASH) with the following parameters: TR/TE = 75/5 ms; FOV = $30.0 \times 30.0 \times 1.57$ mm^3; matrix = 200 × 200 × 45; resolution = 150 × 150 µm; slice direction = transaxial; and NEX = 1. The scanning time was 6 min 33 sec.

Intravenous injection of M-PTX NPs in a mouse model implanted with human oral cancer

OSC-19 cells that had been transfected with luciferase-encoding vector were implanted into the back of Balb/c Slc-nu/nu mice (4–5 weeks old; 4 mice/group) (SLC, Shizuoka, Japan) to create a human oral cancer model. The tumors were allowed to grow to a size of 3–5 mm, and then M-PTX or PTX (12 mg/kg per mouse) were injected daily via a tail vein for 7 days. The tumor volume ratio was calculated by dividing the volume of each tumor by the baseline volume every other day. After 14 days, tumors were harvested, and pathologically examined by hematoxylin-eosin staining (HE) and chemical iron staining.

Magnetically guided focal delivery enhanced the anti-cancer effect of M-PTX *in vivo*

The photon flux was measured once a week for 19 days, as previously described [9]. Regression rate was calculated using the following formula:

Regression rate (%) = Photons/Photons (day0) × 100

Data analysis and statistics

Two-way ANOVA was used to examine in the animal studies in supplemental material Figure 6 and 8. Statistical significance was set at the 0.05 level.

REFERENCES

1. Delley B. From molecules to solids with the DMol3 approach. J Chem Phys. 2000; 113:7756–7764.
2. Becke AD. Density-functional thermochemistry. III. The role of exact exchange. J Chem Phys. 1993; 98:5648–5652.
3. Lee C, Yang W, Parr RG. Development of the Colle-Salvetti correlation-energy formula into a functional of the electron density. Phys Rev B. 1988; 37:785–789.
4. Baker J. Techniques for geometry optimization: A comparison of cartesian and natural internal coordinates. J Comput Chem. 1993; 14:1085–1100.
5. Auckenthaler T, Blum V, Bungartz HJ, Huckle T, Johanni R, Krämer L, Lang B, Lederer H, Willems PR. Parallel solution of partial symmetric eigenvalue problems from electronic structure calculations. Parallel Comput. 2011; 37:783–794.
6. Delley B. A scattering theoretic approach to scalar relativistic corrections on bonding. Int J Quantum Chem. 1998; 69:423–433.
7. Goodenough JB. Theory of the Role of Covalence in the Perovskite-Type Manganites [La, M(II)]MnO3. Phys Rev. 1955; 100:564–573.
8. Moela P, Motadi LR. RBBP6: a potential biomarker of apoptosis induction in human cervical cancer cell lines. Onco Targets Ther. 2016; 9:4721–4735.
9. Ohtake M, Umemura M, Sato I, Akimoto T, Oda K, Nagasako A, Kim JH, Fujita T, Yokoyama U, Nakayama T, Hoshino Y, Ishiba M, Tokura S, et al. Hyperthermia and chemotherapy using Fe(Salen) nanoparticles might impact glioblastoma treatment. Sci Rep. 2017; 7:42783.
10. Eguchi H, Umemura M, Kurotani R, Fukumura H, Sato I, Kim JH, Hoshino Y, Lee J, Amemiya N, Sato M, Hirata K, Singh DJ, Masuda T, et al. A magnetic anti-cancer compound for magnet-guided delivery and magnetic resonance imaging. Sci Rep. 2015; 5:9194 .
11. Eguchi H, Iwatsubo K, Ishikawa Y. Isoform-Selective Regulation of Adenylyl Cyclase by Forskolin Derivatives:Prediction of Selectivity by Computer-Based Analysis. Let Drug Des Discov. 2007; 4:434–441.

Supplementary Table 1: Fe-O-Fe (Goodenough-Kanamori-Anderson) angle for M-PTX A, M-PTX B, M-PTX C, and M-PTX D

	M-PTX A	M-PTX B	M-PTX C	M-PTX D
Fe-O-Fe (Goodenough-Kanamori-Anderson) Angle (°)	128.257	140.768	131.768	171.237

The M-PTX A shows the highest ferromagnetism, having a GKA angle of 128.257°, and it might be chemically stable from the viewpoint of HOMO - LUMO gap and binding energy. On the other hand, M-PTX D has a GKA angle of 171.237°, possessing the weakest ferromagnetic strength, and being almost antiferromagnetic. M-PTX B has a GKA angle of 140.768°, which corresponds closely to the angle of free Fe(Salen) (146.359°) [10]. Hence, in this study, M-PTX B was chosen for further studies based on its reasonable magnetic strength and chemical stability.

Supplementary Table 2: HOMO-LUMO gaps of M-PTX A, M-PTX B, M-PTX C, and M-PTX D

	M-PTX A	M-PTX B	M-PTX C	M-PTX D
HOMO-LUMO gap (eV)	0.693	0.166	0.497	0.851

The chemical stability or reactivity of a M-PTX molecule can be predicted from the HOMO-LUMO gap [11]. The calculated size of the HOMO-LUMO gap decreases in the order of M-PTX D, M-PTX A, M-PTX C, and M-PTX B. As a smaller HOMO-LUMO gap is associated with greater reactivity and lower stability, M-PTX B is most reactive, while M-PTX D is most stable.

Supplementary Table 3: Binding energies of M-PTX A, M-PTX B, M-PTX C, and M-PTX D

	M-PTX A	M-PTX B	M-PTX C	M-PTX D
Binding energy (eV/atom)	–5.128	–5.082	–5.093	–5.109

Binding energy was calculated as described [11]. As lower binding energy corresponds to greater stability, the order of stability is M-PTX A (most stable), M-PTX D, M-PTX C, M-PTX B (least stable).

Supplementary Table 4: Examination of lethal dose and systematic side-effects of M-PTX after injection into mice

A

Group	Dose (mg/kg)	Dead/Total	% Dead
1	100	0/3	0
2	150	1/4	25
3	200	4/6	66.7

B

M-PTX Dose (mg/kg)	Blood examination results				
	Cre (mg/dL)	Tbil (mg/dL)	AST (U/L)	ALT (U/L)	LD (U/L)
200	0.15 ± 0.01	0.10 ± 0.00	58 ± 19.09	25 ± 0.71	462 ± 231.93
100	0.16 ± 0.00	0.10 ± 0.00	43 ± 0.71	24 ± 1.41	355 ± 115.97
Control	0.11 ± 0.01	0.10 ± 0.00	48 ± 1.41	20 ± 7.07	261 ± 53.03

(A) *In vivo* toxicity was examined by evaluating body weight loss, food consumption, and liver and renal functions at 1–2 days and 5–7 days after intravenous M-PTX injection (0, 100, 150, 200 mg/body) into mice. Clinical signs, including activity, were also evaluated. The lethal dose was estimated to be more than 100 mg (n = 3–6).
(B) Changes in parameters of renal function (creatinine), liver function (T-Bil, AST, ALT), and lactate dehydrogenase (LD) of mice were followed for 7 days after M-PTX injection (0, 100, 200 mg/body). Blood chemistry values showed no significant change in any group (n = 2–3).

Supplementary Movie 1: Trapping of M-PTX particles by the magnetic force generated by a permanent magnet in a simulated blood (water) stream. See Supplementary_Movie_1

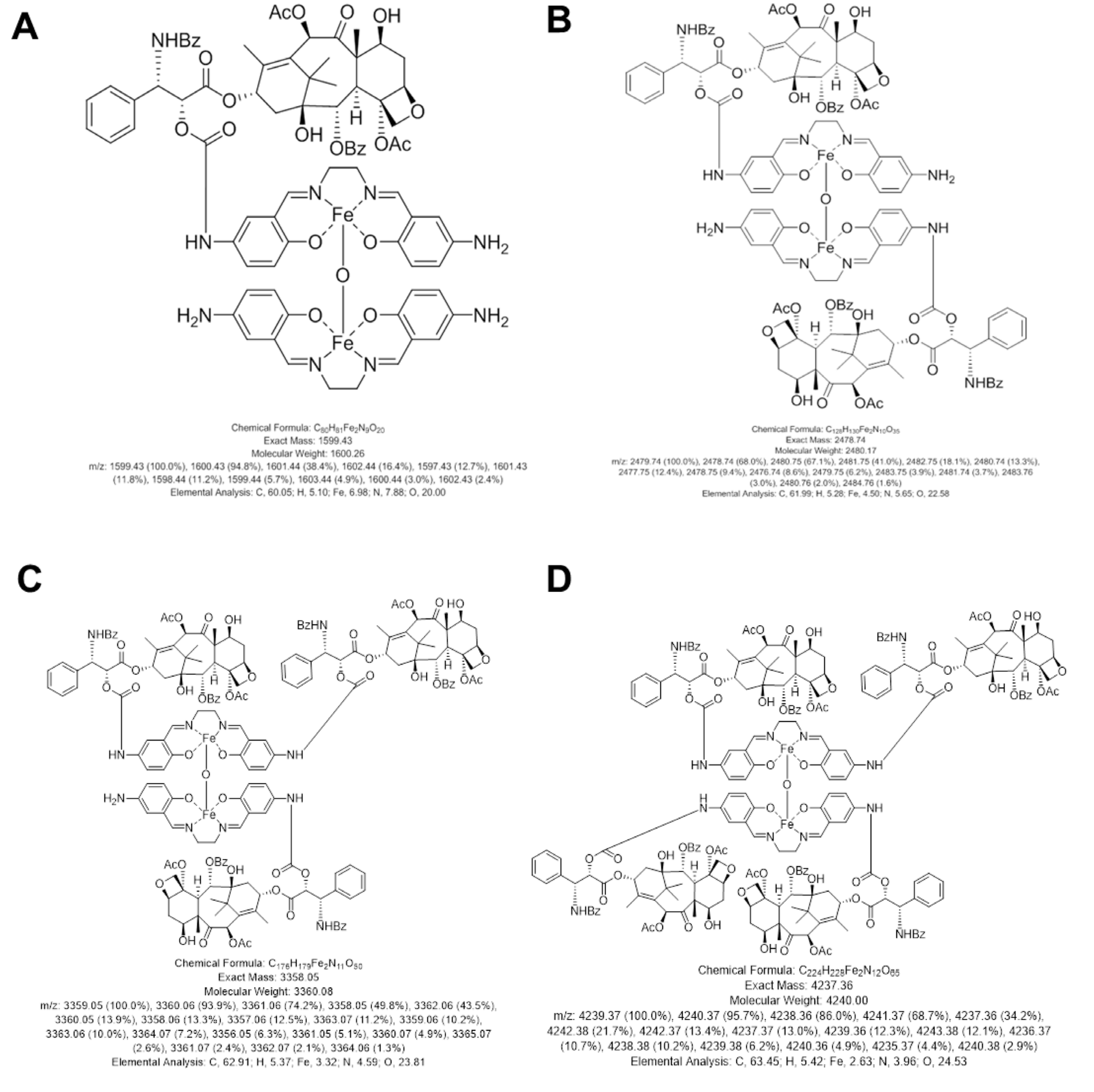

Supplementary Figure 1: Chemical structures of M-PTX. (**A**) M-PTX A: one PTX bound to Fe(Salen). The molecular weight is 1600.26. (**B**) M-PTX B: two PTXs bound to Fe(Salen). The molecular weight is 2480.17. (**C**) M-PTX C: three PTXs bound to Fe(Salen). The molecular weight is 3360.08. (**D**) M-PTX D: three PTXs bound to Fe(Salen). The molecular weight is 4240.00.

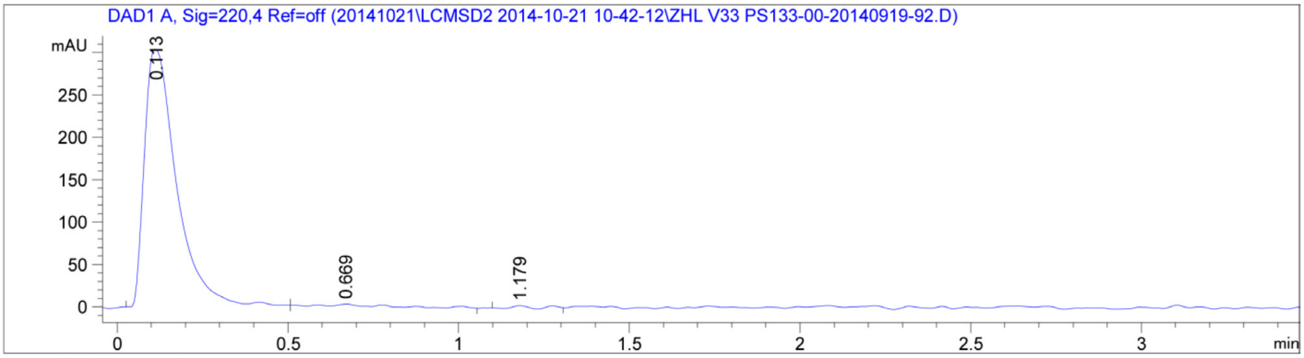

Supplementary Figure 2: HLPC Chart of M-PTX (Fe(Salen):PTX=1:2). Prepared M-PTX was analyzed by HPLC (0.1% formic acid, 25%–97% CH_3CN over 8 min, C18 column 150 × 4.6 mm, 5 µm particle size). The retention time was 0.113 min (96.4%).

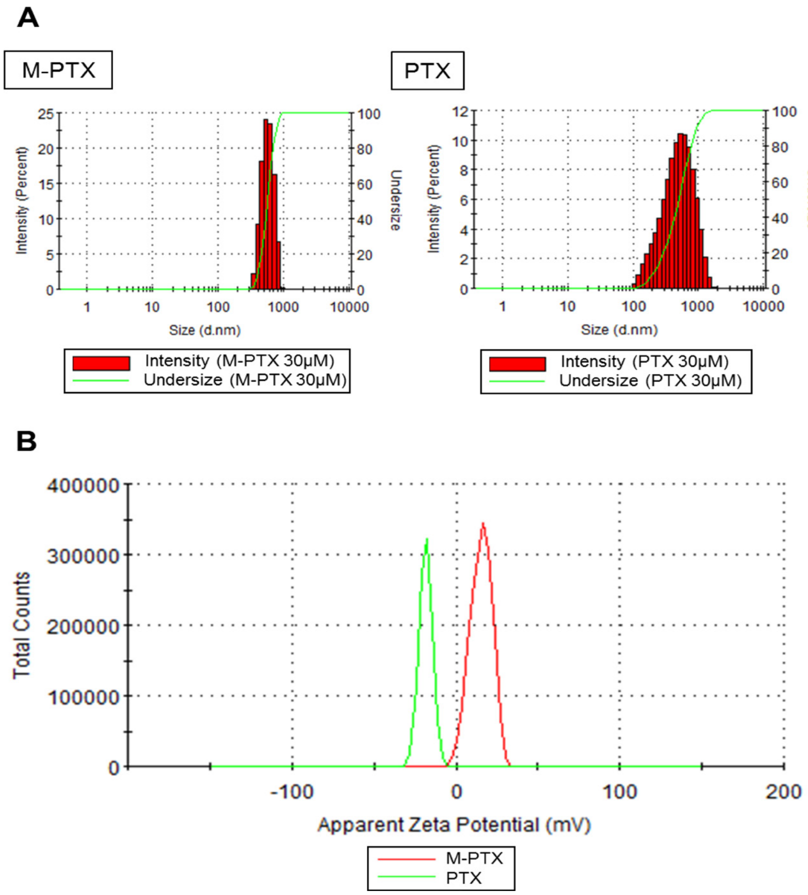

Supplementary Figure 3: Colloidal size and stability of sonicated M-PTX NPs. (**A**) Dynamic light scattering (DLS) histograms of M-PTX NPs and PTX NPs dispersion, showing uniform size distributions of ~551.4 nm (polydispersity index = 0.268) and 571 nm (polydispersity index = 0.552), respectively. (**B**) Zeta potentials of M-PTX NPs and PTX NPs were +15.2 mV and –18.5 mV, indicating fair colloidal stability in aqueous solution.

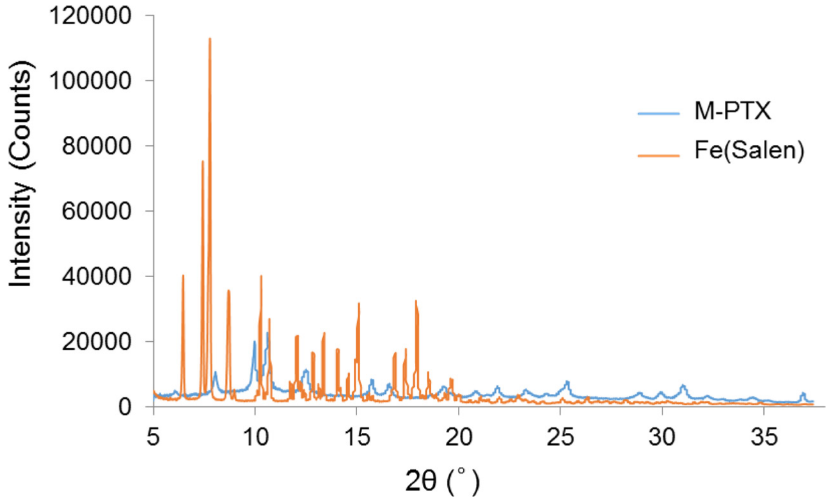

Supplementary Figure 4: Powder XRD characterization of M-PTX B NP in comparison with free Fe(Salen). XRD analysis of M-PTX powder showed distinct peaks due to crystalline PTX, with no marked impurity peaks associated with iron oxide particles. This result confirms covalent linking of Fe(Salen) and PTX, and indicates that the magnetic property of M-PTX is intrinsic, not due to contaminating magnetite.

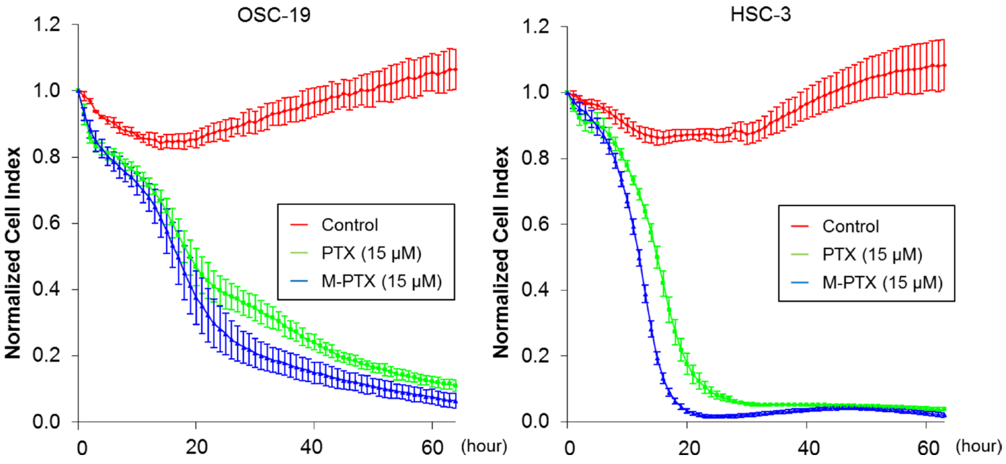

Supplementary Figure 5: Continuous monitoring of cell growth in the presence of M-PTX NPs and PTX. *In vitro* cell proliferation was measured using the xCELLigence (ACEA Biosciences, California, USA) real-time cellular analysis system. M-PTX NPs and PTX showed growth-inhibitory effects on OSC-19 and HSC-3 cells with similar time-dependence.

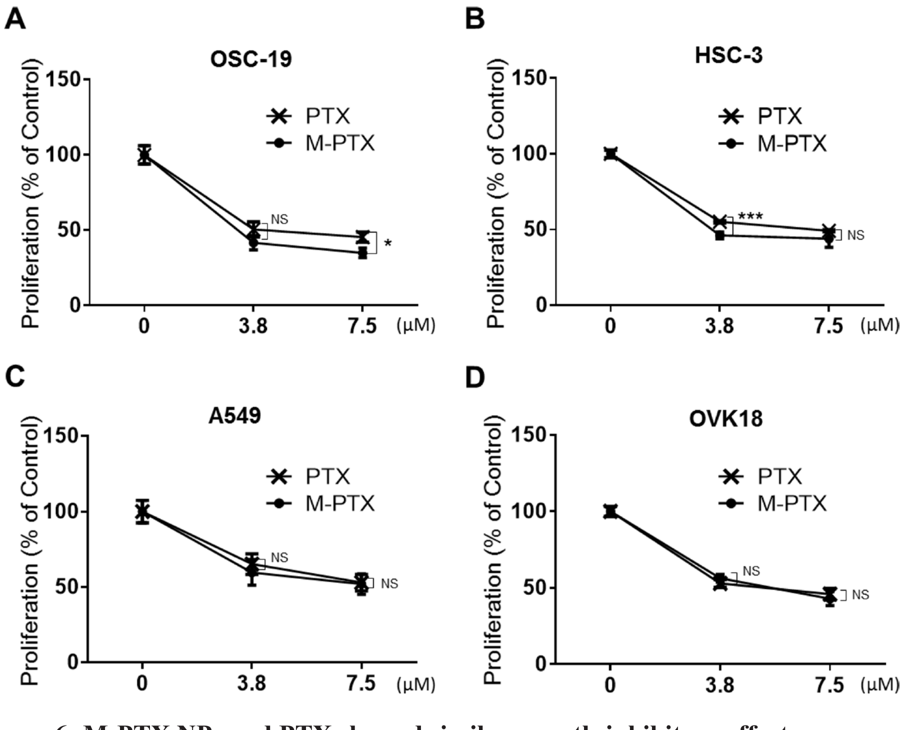

Supplementary Figure 6: M-PTX NPs and PTX showed similar growth-inhibitory effects on cancer cell lines. Growth-inhibitory effects of M-PTX NPs and PTX on OSC-19, HSC-3, A549 (human alveolar adenocarcinoma cell line), and OVK18 (ovarian cancer cell line) cells were evaluated. M-PTX NPs had slightly greater growth-inhibitory effects than PTX ($n = 4$, NS, not significant, $^{*}p < 0.05$, $^{***}p < 0.001$).

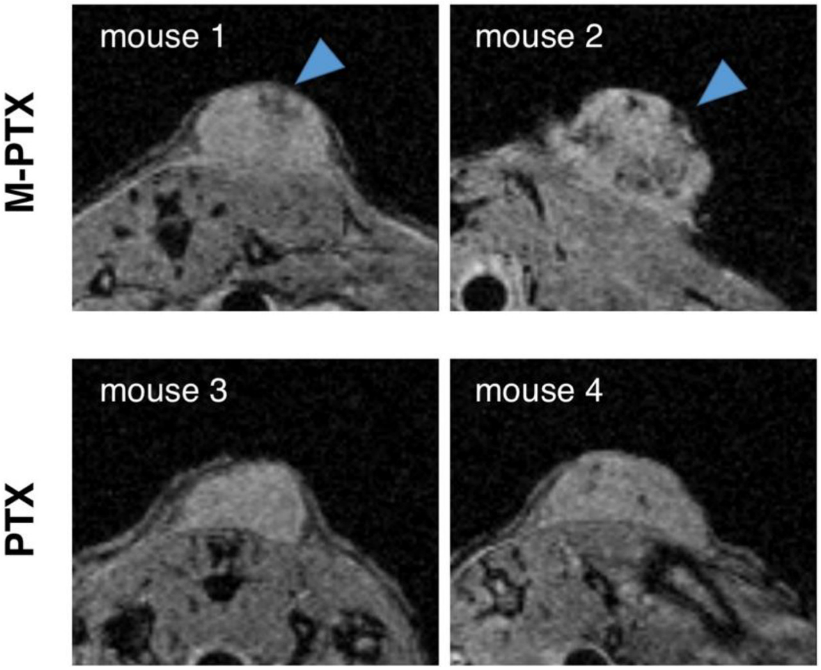

Supplementary Figure 7: Typical *in vivo* MR imaging of the tumor region in M-PTX or PTX administered mice. Signal reduction on T2*-weighted MRI was observed (arrows) in the OSC-19 human carcinoma skin-grafted mice model treated with magnet-guided M-PTX (mouse 1 and 2), or with PTX (mouse 3 and 4). Representative photos are shown.

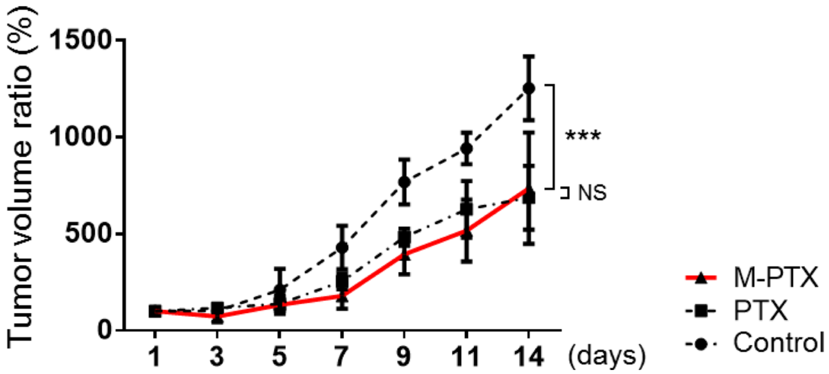

Supplementary Figure 8: M-PTX suppresses tumor growth in mice similarly to commercial PTX. Regression rate based on manual measurement of tumor volume changes (*control vs. PTX vs. M-PTX*) on the back of mice. The red line indicates M-PTX ($n = 4$, NS, not significant, $^{***}p < 0.001$). M-PTX or PTX (12 mg/kg per mouse) were injected from the tail vein for 7 days repeatedly. M-PTX showed a similar anti-cancer effect to that of PTX.

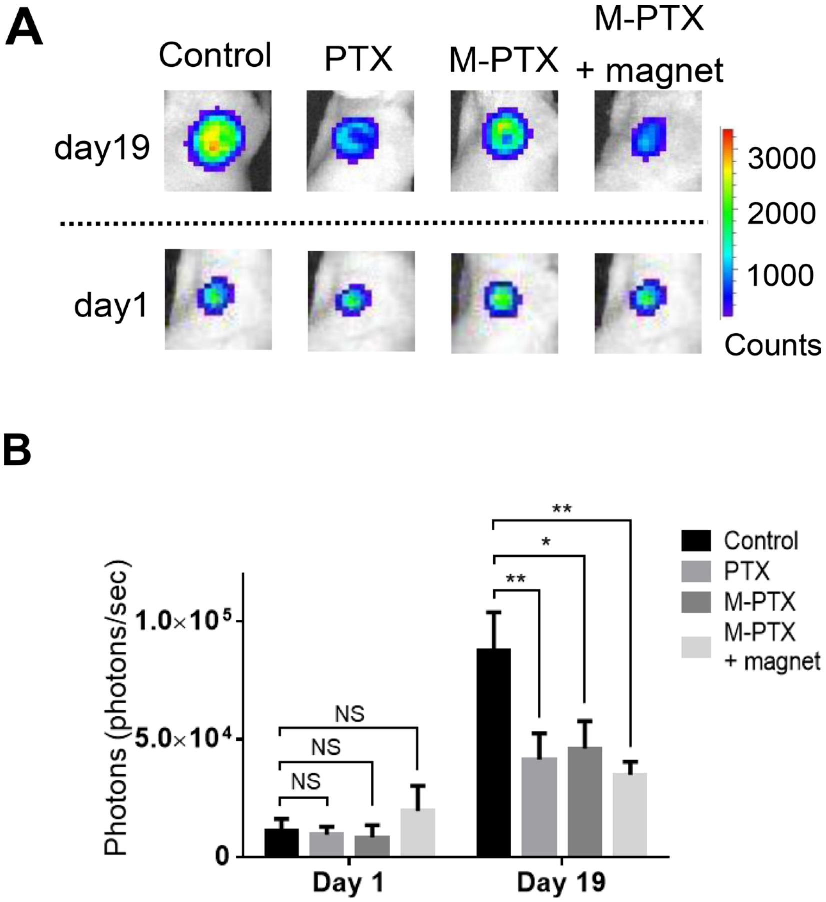

Supplementary Figure 9: Magnet enhances anti-cancer effect of M-PTX in mice. (**A**) IVIS images of mouse back tumor in each treatment group. Control (saline), PTX, M-PTX and M-PTX with permanent magnet. IVIS images of mouse back tumor in each treatment group on Day 19 (*upper*) and Day 1 (*lower*). (**B**) Luminescence intensity in each group is shown at Day 19. (n = 7, NS, not significant, $^{*}p < 0.05$, $^{**}p < 0.01$).
